# Supplementary material for: Health and well-being after being deployed in a major incident; how do Swedish ambulance nurses perceive their health recover process? A qualitative study
Source: BMJ Open. 2023 Jul 4;13(7):e071848. doi: 10.1136/bmjopen-2023-071848 (PMC10335437; doi:10.1136/bmjopen-2023-071848)
Supplement: Author's manuscript [file bmjopen-2023-071848.draft_revisions.pdf]

BMJ Open

BMJ Open is committed to open peer review. As part of this commitment we make the peer review history of every article we publish publicly available.

When an article is published we post the peer reviewers' comments and the authors' responses online. We also post the versions of the paper that were used during peer review. These are the versions that the peer review comments apply to.

The versions of the paper that follow are the versions that were submitted during the peer review process. They are not the versions of record or the final published versions. They should not be cited or distributed as the published version of this manuscript.

BMJ Open is an open access journal and the full, final, typeset and author-corrected version of record of the manuscript is available on our site with no access controls, subscription charges or pay-per-view fees (http://bmjopen.bmj.com).

If you have any questions on BMJ Open's open peer review process please email
info.bmjopen@bmj.com

BMJ Open

Perceived health and the recovery process after being deployed as an ambulance nurse in a major incident- a qualitative study

Journal:	*BMJ Open*
Manuscript ID	bmjopen-2023-071848
Article Type:	Original research
Date Submitted by the Author:	12-Jan-2023

Complete List of Authors:	Blomberg, Karin; Örebro University, Faculty of Medicine and Health<br>Hugelius, Karin; Örebro University, Faculty of Medicine and Health

Keywords:	ACCIDENT & EMERGENCY MEDICINE, Health & safety < HEALTH SERVICES ADMINISTRATION & MANAGEMENT, QUALITATIVE RESEARCH

SCHOLARONE™ Manuscripts

I, the Submitting Author has the right to grant and does grant on behalf of all authors of the Work (as defined in the below author licence), an exclusive licence and/or a non-exclusive licence for contributions from authors who are: i) UK Crown employees; ii) where BMJ has agreed a CC-BY licence shall apply, and/or iii) in accordance with the terms applicable for US Federal Government officers or employees acting as part of their official duties; on a worldwide, perpetual, irrevocable, royalty-free basis to BMJ Publishing Group Ltd ("BMJ") its licensees and where the relevant Journal is co-owned by BMJ to the co-owners of the Journal, to publish the Work in this journal and any other BMJ products and to exploit all rights, as set out in our licence.

The Submitting Author accepts and understands that any supply made under these terms is made by BMJ to the Submitting Author unless you are acting as an employee on behalf of your employer or a postgraduate student of an affiliated institution which is paying any applicable article publishing charge ("APC") for Open Access articles. Where the Submitting Author wishes to make the Work available on an Open Access basis (and intends to pay the relevant APC), the terms of reuse of such Open Access shall be governed by a Creative Commons licence – details of these licences and which Creative Commons licence will apply to this Work are set out in our licence referred to above.

Other than as permitted in any relevant BMJ Author's Self Archiving Policies, I confirm this Work has not been accepted for publication elsewhere, is not being considered for publication elsewhere and does not duplicate material already published. I confirm all authors consent to publication of this Work and authorise the granting of this licence.

For peer review only

Perceived health and the recovery process after being deployed as an ambulance nurse in a major incident- a qualitative study

Authors:

Karin Blomberg, Faculty of Medicine and Health Sciences, Örebro university, Örebro, Sweden.

Karin hugelius*, Faculty of Medicine and Health Sciences, Örebro university, Örebro, Sweden.

* Corresponding author

Corresponding author contacts:

Karin Hugelius
E-mail: karin.hugelius@oru.se
Phone: +46 70 219 59 35

Word count: 4099

For peer review only

ABSTRACT

Objectives: To explore health problems and the recovery process after being deployed in a major incident.

Design: Qualitative, explorative design.

Setting: Prehospital major incidents in Sweden.

Participants and methods: Semi-structured, individual two-session interviews with 15 ambulance nurses were conducted. Data were analysed with thematic analysis.

Results: Being deployed in major incidents was straining and led to both physical health problems and distress. To recover, the ambulance nurses strived to use strategies to distance themselves from the situation and created supportive conditions for their recovery, and if successful, the experiences led to both professional and personal growth and self-awareness. However, being deployed in major incidents without significant preparedness or experience could harm individuals and, in the worst case, end their career.

Conclusions: A successful recovery from the physical and mental exhaustion experienced after being deployed in a major incident required both individual abilities and self-care strategies as well as a supportive working environment. Supporting individual recovery strategies and following up on physical and mental well-being over time should be part of all ambulance services procedures after major incidents.

Keywords: ambulance personnel, ambulance nurses, major incident, resilience, stress, work-related stress, recovery

ARTICLE SUMMARY

Strengths and limitations of this study

- The two-session interviews used stimulated a deeper reflection and rich material to analyse.
- Information power was considered as sufficient.
- Data were analysed and confirmed by researchers with different perspectives on the topic.

- It can never be excluded that another study sample, the characteristics of the major events forming the basis of this study, or the timing of the data collection might have influenced the results.

BACKGROUND

Ambulance personnel are, daily, subject to situations that can evoke distress, but also occasional events such as major incidents, mass casualty situations or disasters that might adversely affect their well-being [1 2]. Responding to major incidents, such as large accidents, terrorist attacks or natural disasters, is a challenging experience even for experienced and trained professionals. Previous studies suggest that such experiences can have both physical and psychological health consequences among first responders [3-5]. After the 9/11 terrorist attack, musculoskeletal problems, breathing problems and undefined bodily pain were reported among the first responders [5]. Among mental

health problems, post-traumatic stress disorder (PTSD) seems to be the most frequently studied condition, and the risk for PTSD has been found to be higher among ambulance personnel than in the general population [3 6]. In particular, professionals in a command position during a major incident or disaster have been found to have an increased risk for mental health problems [3].

Resilience has been described as the ability to “bounce back” after a displacement [7]. It does not necessarily mean the absence of stress reactions but that the person recovers, can carry on with important parts of their life, and can sometimes to grow from these experiences [8]. Resilience should not be seen as an individual process but as a process that is highly dependent on the setting around the person, such as social network, work organizational perspectives, and cultural and community aspects [8]. Being resilient does not necessarily mean the absence of health problems or pathology but being able to cope with the situation and actively adapt in a healthy way [9]. The resilience of ambulance nurses after being deployed in major incidents is concerning from many perspectives: for the individual affected, from an employer perspective and for the broader community that relies on their services. Especially in the aftermath of a major incident, the medical resources might be stretched, and therefore, it is of extra importance that ambulance nurses are resilient and stay healthy after such events. However, the knowledge on how to promote resilience among ambulance personnel after being deployed in major incidents is still limited. Therefore, this study aimed to explore ambulance nurses’ perceived health and the recovery process after being deployed in major incidents.

METHODS

Design

An explorative, qualitative study was conducted.

Study setting

The study was conducted in Sweden. In XXX, all emergency ambulances are staffed with at least one registered nurse, most often specialised in prehospital emergency care (called ambulance nurse) and one paramedic or a second nurse. In this study, a major incident was defined as an incident that forced the health care system to heighten the level of response due to the event.

Study sample and recruiting

Nurses or specialized nurses within the ambulance services were recruited by invitations via social media, in official or private groups specifically focused on Swedish ambulance personnel, and by invitations distributed through ambulance managers in health care regions where a major incident had occurred during the last five years. Inclusion criteria were having at least one year of working experience as a nurse within the ambulance services and having authentic experiences from being deployed in a major incident during the last five years. Exclusion criteria were personal or professional relationships with any of the authors. Eligible participants who wanted to participate in the study were asked to read full study information posted on a web page and to fill in a form with their contact information. All nurses who showed interest were contacted by a researcher (XX) via phone to set up a time for the interview. A total of 15 nurses responded to the invitation and were included in the study. No one who volunteered to participate was excluded.

Data collection

For peer review only

Two-session individual interviews [10], (n = 27) were conducted face to face (n = 2) or by phone (n = 25) with 15 nurses. All the interviews were conducted by one researcher (KH) during the period of December 2018 to February 2020 using a semi-structured interview guide. The interview guide covered questions on experiences working in a major incident, preparations for such experiences, perceived health effects and recovery strategies after such experiences. The interview guide was tested in two pilot interviews, after which only minor corrections were made. The pilot interviews were therefore included in the analysis. The study participants were asked to focus on one specific major incident, even though many of the participants expressed experiences gained from several major incidents. Probing questions, for example, “What do you mean by that…” or “Can you further describe…”, were used to obtain a deeper understanding of their experiences. The second interview was conducted within 14 days of the first interview and aimed to follow up on the dialogue but also to follow up on the participant’s well-being. Three participants declined a second interview due to personal circumstances such as a planned vacation, which resulted in a data set of 27 interviews in total. All interviews were audio-recorded and transcribed verbatim by a professional transcriber. The COREQ checklist was used to report the study.

Analysis

An inductive thematic analysis was used to analyse the data [11]. After transcription, the whole texts were read through several times to get familiar with the data, as the researcher (KB) primarily responsible for the analysis was not involved in data collection. Thereafter, meaning units responding to the aim of the study were extracted and coded by the first author (KH). The codes were thereafter clustered into sub-themes and themes reflecting both the coded extracts and the entire data set. This clustering formed a broader understanding of patterns within the phenomenon studied. During this phase, two researchers participated (KB, KH). A final naming of the themes and sub-themes was determined during discussions between the two researchers. Finally, the results were formulated, and quotations were selected to illustrate the findings and to increase trustworthiness.

Ethical considerations

All participants had received written and verbal study information that their participation was voluntary and that they had the right at any time to refrain from further participation without specifying any reasons. Before the first interview, informed consent was obtained. Because major incidents are rare in Sweden, no information on the study participants or the exact situation behind the specific quotation was provided to protect the participants’ identity. To detect signs of potential traumatization or need for professional counselling, all study participants were asked to respond to the Posttraumatic Checklist-Civilian (PCL-IV), Swedish version. A cut-off value of over 40 was an indicator for remaining stress [12]. The research group had experience and competence to refer a study participant for further support if such a need arose. The study was approved by the Swedish Ethical Review Authority (reference number2018:232).

Patient and Public Involvement

No patient involved.

RESULTS

Overall, 15 ambulance nurses, participated in the 27 interviews. The interviews lasted between 2 and 89 minutes (first interviews 24 to 89 minutes, median 52 minutes; second interviews 2 to 22 minutes,

For peer review only

median 5 minutes). Of the study participants, ten were males and five females, aged 29 to 62 years old (median age 41), with professional experience in ambulance services varying from 1 to 39 years (median 18 years). The major incidents covered in the study included traffic accidents, fires, terrorist attacks and explosions. Demographics, professional experiences, type of major incident and the time for each interview are shown in Table 1. None of the participants screened indicated remaining traumatic stress according to the PCL-IV.

Table 1. Overview of study participants' (n = 15) demographics, type of incident and times for the interviews

Parti-cipant	Sex	Age	Profession	Experience within the ambulance services (years)	Type of incident in focus for the interview	Minutes of interview 1/ Minutes of interview 2
1	Male	37	Specialized nurse	16	Bridge collapse	89/10
2	Female	41	Specialized nurse	19	Traffic accident	87/12
3	Male	62	Specialized nurse	39	Terrorist truck attack	55/12
4	Male	38	Nurse	22	Fire in an elderly home	65/5
5	Male	51	Specialized nurse	29	Bus accident	48/0
6	Male	38	Specialized nurse	18	Bus accident	76/10
7	Male	51	Nurse	26	Bridge collapse	45/0
8	Male	36	Nurse	12	Explosion	74/22
9	Female	29	Nurse	3	Terrorist truck attack	52/16
10	Female	52	Nurse	17	Bus accident	45/0
11	Female	29	Specialized nurse	1	Terrorist truck attack	24/5
12	Female	32	Specialized nurse	18	Train-bus collision	51/4
13	Male	41	Specialized nurse	6	Bus accident	67/0
14	Male	61	Specialized nurse	29	Traffic accident	37/12
15	Male	43	Specialized nurse	11	Chemical incident	25/5

Table 2 provides an overview of the main theme and sub-themes.

Table 2. Overview of the findings

**Main themes**	**Being exhausted and vulnerable**	**Getting distance from the situation**	**Establishing supportive conditions for recovery**	**Personal and professional development**
**Sub-themes**	Experiencing physical health problems	Using self-reflection	Getting the whole picture	Professional growth
	Experiencing mental health problems	Transforming from professional to personal	Creating social support	Personal growth
	Feeling vulnerable		Experiencing professional support	Failure to cope and recover

For peer review only

The experiences of health problems and recovery after being deployed in a major incident could be described as a process staring right after the deployment with being physically and mentally exhausted and vulnerable, continuing with a phase of striving to find strategies that provided distance from the situation and supportive conditions for recovery, followed by an incorporation of the experiences into both personal and professional development (see Figure 1).

--INSERT Figure 1 about here--

Figure 1. The process of recovery after deployment in a major incident.

Being exhausted and vulnerable

After finalizing the deployment in the major incident, all nurses described feelings of being totally exhausted, both physically and mentally. Physical health problems such as a general bodily pain, headache or dizziness were most intense during the first few days. A tiredness that was overwhelming was described; at the same time, sleeping problems could be present. The nurses described that their minds and thoughts were constantly busy processing impressions from the major incident, as a kind of background music, making it hard to relax. Additionally, cognitive problems, such as having difficulties concentrating, were reported. Most mental reactions started after some time and were more intense a few days after the incident.

> "I was so tired. Extremely tired. Had some pain in my body. Needed to rest. And then…the thought went like in a loop- coming back all the time; what you saw, what you did. I kind of went through the situation all over again and again. It lasted for some day maybe. Then I could move forward and put it behind me."

The experience of being exhausted was accompanied by feelings of being vulnerable. Being faced with how the incident had caused personal tragedies and affected human people reminded them of the fragility of life and made the nurses feel vulnerable.

> "But then…. after a while. It was very hard actually. But it took three days until I could take it in: those things I've seen there in the bus. I read about the victims in a newspaper. Their families spoke about them, of course. Then I felt….it was tough…I became sad. "

The nurses sometimes put themselves in the position of the injured or family members, as if the incident had affected themselves or their own lives. That increased feelings of being sad and powerless. Sometimes, existential thoughts occurred, such as questioning the meaning of life or the meaning of the incident that had occurred.

Getting distance from the situation

After having overcome the exhaustion that was experienced in the first phase, the nurses strived to get distance from the major incident. For some, this phase started only a few hours after the incident, whereas for others, it could be some days after the incident. Being alone, taking one's time to reflect on the actual incident as well as one's own emotions and thoughts, and finding one's own "inner place" were essential and contributed to decreased physical problems as well as decreased distress. Another strategy used to get distance from the situation was to return as soon as possible to

For peer review only

everyday life and routines, both personal and professional. Such activities and contexts included everyday practices such as eating together and watching TV with colleagues, including those they had worked with during the major incident, as a method of “normalization”. Using humour, for example, giggling together about other things that had occurred during the incident, gave them distance from the experiences.

> “We went back to the [ambulance] station and then we said that we need a break now, to catch up with ourselves. We needed some time off, without new calls. You cannot just keep on going, it’s simply not possible always. I took a long, hot shower. It is a good way to recover, you defrost yourself, and kind of wash the event off yourself. I think it works. Then we made some tea and had a sandwich and just sat in the sofa watching TV.”

Several of the nurses described activities that could be understood as rituals to handle the transition from being a professional during the major incident to becoming a private person. An example of such rituals was taking a bath or shower to cleanse oneself of the experienced situation.

> “They had made sandwiches and so, but I could not eat. My body was on high alert so to say, all I wanted was to take a shower, to wash the incident off myself, to clean myself from it in some way…. I did not want to talk about it actually…. “

Most of the nurses claimed that they preferred to stay with their colleagues and to keep on working after the major incident, even if offered to go home immediately. Staying at work also contributed to normalization and de-dramatized the situation.

Establishing supportive conditions for recovery

A significant part of the individual responsibility of each nurse was to create good conditions for their own recovery. However, the preferred conditions varied widely. A common understanding was that stress reactions were to be seen as normal but still required quite some time to recover from. Participants with less professional experience described how they were unprepared for the time and energy required to recover from working during major incidents. Creating their “own story” of the incident and their efforts sometimes required information from other perspectives or persons involved. One example of such information needed could be information about the victims’ outcome.

> “Well, for me, I had the whole picture, from alarm to the end so to say, but many of our colleagues had only arrived and picked up a patient they took to the hospital. And they needed to get information on what had happened before they arrived….and I needed information on what had happened to the patient during the transport. So, we spend some time to talk about these things, to clear the scenario out for everyone”

If there were fatalities, some nurses conducted private rituals, such as lighting a candle for the victims. Such rituals could serve as an endpoint of the major incident. Creating possibilities of informal sharing of experiences with others who understood the context, such as colleagues or first-line managers, was a crucial part of the supportive environment. Such sharing also contributed to a feeling of being part of the team and getting confirmation of one’s efforts during the major incident.

> “I think that it is very important with the colleagues, especially those who have long experience, who have seen most things and you can say anything- they can bear to

For peer review only

> hear it. Because they have seen it themselves, some time. I cannot tell anyone else; they will not understand, and they will distance themselves. I know that my colleagues understand when I have to make a joke about something horrible, because there is no other way to cope with it…That is a respect for the human being as I don't recognize from any other profession."

Almost all nurses reflected on individual self-reflection and informal social support compared with organized psychosocial support, such as after-action reviews or group sessions including psychological debriefing. Many of the nurses considered such organized sessions not to be supportive of their recovery, and for some, they had an opposite effect than intended. Additionally, individuals who did not feel like talking or being around colleagues at all had to be respected. A general caring attitude from colleagues and first-line managers was important to create trust that if someone needed professional support, such support would be immediately provided. Because most mental reactions occurred after some time following the incident, many participants meant that the concern and care from colleagues and managers also had to be present after some time and that offers of professional support were more valuable after some time than during the acute phase.

Personal and professional development

The experiences gained both from the major incident itself but also from reactions and the following recovery process needed to be integrated into the personal and professional identity and could then contribute to professional and sometimes personal growth. Some nurses stated that this process of incorporating the major event as a part of their overall experiences was a skill that could be learned over years within the emergency medical services. To facilitate this transformation, continuing to take part in ordinary work, accepting the distress and relying on the individual recovery process were supportive. Senior colleagues and managers could play an important role in this process by confirming the occurrence of distress and sharing their experiences of similar recovery processes. At the same time as it was perceived as important to finalize and "store" their experiences, the nurses expressed worries about what impact such strong experiences would have on them as both professionals and people in a longer perspective.

> "My fiancé came home and just held me. We didn't talk, it wasn't necessary. He just held me. I thought that I had to quit this job. But at the same time; it is a part of me, who I am. I want to be in situations like this. I want to help. I'm an ambulance nurse, in my person. And so is he. It's part of our lives, that we understand each other's needs in situations like this. I was pregnant that day. He was the only person who knew. I was so worried for the future…."

Over time, new perspectives on life, on being a human and on the role of the emergency medical services developed, mostly adding wisdom and being more resilient, as both a person and as a nurse. Most often, this process was positive, leading to an increased self-awareness and ability to manage and endure severe situations.

> "Some new people that come to us think that it is a tough environment within the ambulance services, that we are hard as stone. I say it is the opposite. We witness a lot, we meet people in all kinds of situations, when they are at most vulnerable, when they are between life or death, when they don't want to live any more, when they are severely beaten…and that could happen to us all. Everyone. Life is not always clean

For peer review only

> and tidy, not always fair. You realize that after some years. We can laugh about it, together, about things that are not really fun for anyone else. Undramatized it. We live today, but you never know about tomorrow… You learn that within the ambulance services. I think it is good. You need that reminder sometimes. And I live a richer life thanks to that."

However, sometimes, the burden of the experiences from the major event was too hard to endure, and the recovery process did not end with a positive development. In such situations, the nurses felt overwhelmed and unsuccessful, which led to persistent physical or mental health problems and in some cases, an ending of their career.

> "My colleague, she was incident commander in an accident where two children died. She left the ambulance services after that. She just cried as soon as she came to the work. It was very hard to see her, actually. She was a very competent ambulance nurse, but this crushed her. I tried, we tried all of us. But she took it very personally, she withered away…"

The risk for such outcomes was experienced as higher if the nurse was unexperienced and lacked proper formal or mental preparedness for being faced with the complexity and demands of a major incident.

DISCUSSION

Being deployed as an ambulance nurse in a major incident led physical- and psychological health problems and feelings of being exhausted and vulnerable. To recover, the ambulance nurses took responsibility and strived to find strategies to gain distance from the situation and to create supportive conditions. If successful, the recovery process led to professional and personal growth. However, being deployed in major incidents without significant preparedness or experience could harm individuals and, in the worst case, end their career.

In this study, no serious physical injuries were reported, but despite that, many participants described physical health problems and exhaustion. Although physical well-being has been found to be an important mediator for psychological wellbeing [8], the physical status of ambulance nurses is important when discussing recovery after working in stressful situations.

The nurses demonstrated a high level of awareness of the occurrence of stress and the risks for being traumatized but also self-knowledge and strategies to manage distress. At the same time, several expressed worries about colleagues and sometimes fear of being exposed to situations that might overload their capacity to recover. A realistic appraisal and active decision-making on how to cope with the situation can contribute to resilience after stressful events [7]. In this study, the participants described different activities that can be seen as "rituals", striving for balance concerning professional boundaries between themselves as a person and being an ambulance personnel. It seems that these "rituals" also mediated a transition from "being in the major incident" to "being after the incident" and moving to a more ordinary everyday life. In the theoretical framework of transition [13], this process has been described as a passage from one phase, condition or status to another or as a period between relative stable phases. For a successful transition process, the person needs to have awareness of both their situation and the transition process, and the level of engagement is essential. The participants in this study expressed a strong commitment and

For peer review only

convention that they were accountable themselves for creating their own recovery process. Being aware of one's own responsibility for one's well-being and using potential traumatic events and severe stress as a foundation to learn and grow from [8] can be applied to the results of this study. All transitions or changes also require time [13], which was strongly emphasized by the study participants who expressed that they sometimes were surprised that their own recovery took longer than expected.

Self-care has been found to be a key to protect against negative health impacts [14]. The study also confirmed that ambulance nurses need to acquire a unique set of personal qualities to promote self-care and self-resilience in their everyday work [15]. A question raised was whether the competence to develop self-care strategies and use experiences to grow is something that can developed over time or whether it is personal attribute needed to be comfortable in a profession such as being a nurse within the ambulance services. Is there a self-selection component that can support resilience within the ambulance services, or is it a result of a professional development process over time?

To mitigate the negative health effects and promote physical and mental wellbeing requires an awareness of the demands as well as the recovery process. Prevention of work-related traumatic stress should rely on a sound psychosocial work environment, systematic training of employees, social support from colleagues and managers, and proper follow-up of employees after a critical event [16]. Well-functioning supportive structures and working environment have been suggested to compensate for short professional experiences [14] and might therefore be of extra importance for less experienced ambulance personnel. Social support seems particularly powerful to reduce negative outcomes of stress [14 17 18]. In line with evidence, organized sessions such as debriefings or group counselling were not experiences that contributed to recovery or resilience and should therefore not be part of the organizational support systems [19 20]. Instead, this study emphasizes the employer's responsibility to understand the recovery process, to enable time and space for the recovery, and to systematically and with empathy follow up on both physical and mental health, both in the acute phase and over time. Ensuring that ambulance personnel are "fit for duty" after being deployed in major incidents should therefore be a shared responsibility between the individual professional and the employer as a natural component in a resilient ambulance service both in the every day services and in major incidents.

Limitations

As in all qualitative research studies, it can never be excluded that another study sample, the characteristics of the major events forming the basis of this study, or the timing of the data collection might have influenced the results. However, the study sample size was considered to have information power [21], influenced by recruiting study participants with quite recent experiences, high-quality dialogue during the interviews, the two-session interviews and the use of a systematic analysis methodology. The two-session interview design, chosen to ensure that all data were collected and to ensure the well-being of the study participants, contributed to rich data and to verifying the interpretations of the first interview statements. All data were analysed and confirmed by researchers with different perspectives on the topic, including an extensive preunderstanding of the ambulance services, major incidents and caring in emotionally challenging situations, which was found to contribute to the understanding of the data.

CONCLUSION

For peer review only

The ambulance nurses demonstrated a high level of awareness of the occurrence of stress and individual responsibility to find strategies to recover. A successful recovery from the physical and mental exhaustion experienced after being deployed in a major incident required both individual abilities and self-care strategies as well as a supportive working environment. Organized sessions such as debriefings or group counselling did not contribute to recovery and should therefore not be part of the routine organizational support systems. Instead, supporting individual recovery strategies and following up on physical and mental well-being both in the acute phase and over time should be part of all ambulance services procedures after major incidents to promote individual and organizational resilience.

List of abbreviations

Post-traumatic stress disorder (PTSD)

Posttraumatic Checklist-Civilian (PCL)

Funding

This research received no specific grant from any funding agency in the public, commercial or not-for-profit sectors.

DECLARATIONS

Ethics approval and consent to participate
The study was reviewed and approved by the Swedish Ethical Review Authority (ref id 2018:232).

Availability of data and materials
The data analysed during the current study are not publicly available due to Swedish laws on research ethics and the ethical approval provided by the Swedish Ethical Review Authority.

Competing interests
The authors declare that they have no competing interests.

Author contributions
KB: Conceptualization, analysis, writing of the manuscript, KH: Conceptualization, data collection, writing of the manuscript. Both authors read and approved the final manuscript.

Acknowledgement
The authors would like to thank Dr Samuel Edelbring for his valuable contribution to the initial design and planning of the data collection for this study.

References

1. Halpern J, Maunder RG, Schwartz B, et al. Identifying, Describing and Expressing Emotions After Critical Incidents in Paramedics. *Journal of Traumatic Stress* 2012;25:111-14.
2. Lawn S, Roberts L, Willis E, et al. The effects of emergency medical service work on the psychological, physical, and social well-being of ambulance personnel: a systematic review of qualitative research. *BMC Psychiatry* 2020;20(1):348.

For peer review only

3. Berger W, Coutinho ES, Figueira I, et al. Rescuers at risk: a systematic review and meta-regression analysis of the worldwide current prevalence and correlates of PTSD in resuce workers. *Social Psychiatry and Psychiatric Epidemiology* 2012;47(6):1001-11.
4. Skogstad L, Heir T, Hauff E, et al. Post traumati stress among rescue workers after terror attacks in Norway. *Occupational and Enivornmental Medicine* 2016;66(7):528-35.
5. Smith EC, Holmes L, Burkle FM. The Physical and Mental Health Challenges Experienced by 9/11 First Responders and Recovery Workers: A Review of the Literature. *Prehospital and Disaster Medicine* 2019 Dec;34(6):625-631.
6. Petrie K, Milligan-Saville J, Gayed A, et al. Prevalence of PTSD and common mental disorders amongst ambulance personnel: a systematic review and meta-analysis. *Social Psychiatry and Psychiatric Epidemiology* 2018;53(9):897-909.
7. Southwick SM, Bonanno GA, Masten AA, et al. Resilience definitions, theory, and challenges: interdisciplinary perspectives. *European Journal of Psychotraumatology* 2014;1(5):25338.
8. Southwick SM, Charney DS. Resilience. The Science of Mastering Life´s Greatest Challanges. Second ed.New York: Cambridge University Press 2018.
9. Shakespeare-Finch J, Daley E. Workplace belongingness, distress, and resilience in emergency service workers. *Psychological Trauma: Theory, Research, Practice, and Policy* 2017;9(1):32-35.
10. Read BL. Serial Interviews: When and Why to Talk to Someone More Than Once. *International Journal of Qualitative Methods* 2018; 17.(1).
11. Braun V, Clarke, V. Using thematic analysis in psychology. *Qualitative Research in Psychology* 2006;3(2):77-101.
12. Arnberg F, Michel PO, Johannesson KB. Properties of Swedish Posttraumatic Stress Measures after a Disaster. *Journal of Anxiety Disorders* 2014;28(4):402-09.
13. Meleis AI, Sawyer LM, Im EO, et al. Experiencing transitions: an emerging middle-range theory. *Advancec in Nursing Science* 2000;23(1):12-28.
14. Smith E, Walker T, Burkle FM. Lessons in Post-Disaster Self-Care From 9/11 Paramedics and Emergency Medical Technicians. *Prehospital and Disaster Medicine* 2019;34(3):335-39.
15. Clompus SR, Albarran JW. Exploring the nature of resilience in paramedic practice: A psycho-social study. *International Emergency Nursing* 2016;28:1-7.
16. Skogstad L, Skorstad, M., Lie, A., Conradi, H.S., Heir, T., Weisaeth, L. Work-related post- traumatic stress disorder. *Occupational Medicine* 2013;63(3):175-82.
17. Behnke A, Rojas R, Karrasch S, et al. Deconstructing Traumatic Mission Experiences: Identifying Critical Incidents and Their Relevance for the Mental and Physical Health Among Emergency Medical Service Personnel. *Frontiers in Psychologt* 2019;10:2305.
18. Arnberg F, Hultman CM, Michel M, et al. Social Support Moderates Posttraumatic Stress and General Distress After Disaster. *Journal of Traumatic Stress* 2012;25(6):721-27.
19. Rose SC, Bisson J, Churchill R, et al. Pshycological debriefing for preventing post traumatic stress disorder (PTSD) (Review). Cochrane Database Syst Rev: The Cochrane Library, 2006.
20. International Society for Traumatic Stress Studies. Posttraumatic Stress Disorder Prevention and Treatment Guidelines. Methodology and Recommendations. 2019. Avalibe at: https://istss.org/getattachment/Treating-Trauma/New-ISTSS-Prevention-and-Treatment-Guidelines/ISTSS_PreventionTreatmentGuidelines_FNL.pdf.aspx. Accessed: January 09, 2023.
21. Malterud K, Siersma VD, Guassora AD. Sample Size in Qualitative Interview Studies: Guided by Information Power. *Qualitative Health Reseach* 2016;26(13):1753-60.

For peer review only

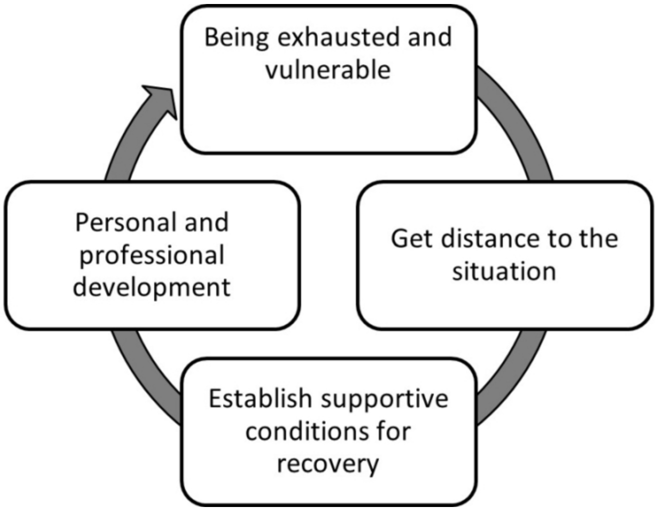

Figure 1. The process of recovery after deployment in a major incident.

107x54mm (300 x 300 DPI)

COREQ (COnsolidated criteria for REporting Qualitative research) Checklist

A checklist of items that should be included in reports of qualitative research. You must report the page number in your manuscript where you consider each of the items listed in this checklist. If you have not included this information, either revise your manuscript accordingly before submitting or note N/A.

Topic	Item No.	Guide Questions/Description	Reported on Page No.
**Domain 1: Research team and reflexivity**			
Personal characteristics			
Interviewer/facilitator	1	Which author/s conducted the interview or focus group?	
Credentials	2	What were the researcher's credentials? E.g. PhD, MD	
Occupation	3	What was their occupation at the time of the study?	
Gender	4	Was the researcher male or female?	
Experience and training	5	What experience or training did the researcher have?	
Relationship with participants			
Relationship established	6	Was a relationship established prior to study commencement?	
Participant knowledge of the interviewer	7	What did the participants know about the researcher? e.g. personal goals, reasons for doing the research	
Interviewer characteristics	8	What characteristics were reported about the inter viewer/facilitator? e.g. Bias, assumptions, reasons and interests in the research topic	
**Domain 2: Study design**			
Theoretical framework			
Methodological orientation and Theory	9	What methodological orientation was stated to underpin the study? e.g. grounded theory, discourse analysis, ethnography, phenomenology, content analysis	
Participant selection			
Sampling	10	How were participants selected? e.g. purposive, convenience, consecutive, snowball	
Method of approach	11	How were participants approached? e.g. face-to-face, telephone, mail, email	
Sample size	12	How many participants were in the study?	
Non-participation	13	How many people refused to participate or dropped out? Reasons?	
Setting			
Setting of data collection	14	Where was the data collected? e.g. home, clinic, workplace	
Presence of non-participants	15	Was anyone else present besides the participants and researchers?	
Description of sample	16	What are the important characteristics of the sample? e.g. demographic data, date	
Data collection			
Interview guide	17	Were questions, prompts, guides provided by the authors? Was it pilot tested?	
Repeat interviews	18	Were repeat inter views carried out? If yes, how many?	
Audio/visual recording	19	Did the research use audio or visual recording to collect the data?	
Field notes	20	Were field notes made during and/or after the inter view or focus group?	
Duration	21	What was the duration of the inter views or focus group?	
Data saturation	22	Was data saturation discussed?	
Transcripts returned	23	Were transcripts returned to participants for comment and/or	

For peer review only

Topic	Item No.	Guide Questions/Description	Reported on Page No.
		correction?	
**Domain 3: analysis and findings**			
Data analysis			
Number of data coders	24	How many data coders coded the data?	
Description of the coding tree	25	Did authors provide a description of the coding tree?	
Derivation of themes	26	Were themes identified in advance or derived from the data?	
Software	27	What software, if applicable, was used to manage the data?	
Participant checking	28	Did participants provide feedback on the findings?	
Reporting			
Quotations presented	29	Were participant quotations presented to illustrate the themes/findings? Was each quotation identified? e.g. participant number	
Data and findings consistent	30	Was there consistency between the data presented and the findings?	
Clarity of major themes	31	Were major themes clearly presented in the findings?	
Clarity of minor themes	32	Is there a description of diverse cases or discussion of minor themes?	

Developed from: Tong A, Sainsbury P, Craig J. Consolidated criteria for reporting qualitative research (COREQ): a 32-item checklist for interviews and focus groups. *International Journal for Quality in Health Care*. 2007. Volume 19, Number 6: pp. 349 – 357

Once you have completed this checklist, please save a copy and upload it as part of your submission. DO NOT include this checklist as part of the main manuscript document. It must be uploaded as a separate file.

For peer review only

BMJ Open

Health and wellbeing after being deployed in a major incident; how do Swedish ambulance nurses perceive their health recover process? A qualitative study.

Journal:	*BMJ Open*
Manuscript ID	bmjopen-2023-071848.R1
Article Type:	Original research
Date Submitted by the Author:	05-Apr-2023
Complete List of Authors:	Blomberg, Karin; Örebro University, Faculty of Medicine and Health<br>Hugelius, Karin; Örebro University, Faculty of Medicine and Health
**Primary Subject Heading**:	Emergency medicine
Secondary Subject Heading:	Nursing, Qualitative research
Keywords:	ACCIDENT & EMERGENCY MEDICINE, Health & safety < HEALTH SERVICES ADMINISTRATION & MANAGEMENT, QUALITATIVE RESEARCH

SCHOLARONE™
Manuscripts

I, the Submitting Author has the right to grant and does grant on behalf of all authors of the Work (as defined in the below author licence), an exclusive licence and/or a non-exclusive licence for contributions from authors who are: i) UK Crown employees; ii) where BMJ has agreed a CC-BY licence shall apply, and/or iii) in accordance with the terms applicable for US Federal Government officers or employees acting as part of their official duties; on a worldwide, perpetual, irrevocable, royalty-free basis to BMJ Publishing Group Ltd ("BMJ") its licensees and where the relevant Journal is co-owned by BMJ to the co-owners of the Journal, to publish the Work in this journal and any other BMJ products and to exploit all rights, as set out in our licence.

The Submitting Author accepts and understands that any supply made under these terms is made by BMJ to the Submitting Author unless you are acting as an employee on behalf of your employer or a postgraduate student of an affiliated institution which is paying any applicable article publishing charge ("APC") for Open Access articles. Where the Submitting Author wishes to make the Work available on an Open Access basis (and intends to pay the relevant APC), the terms of reuse of such Open Access shall be governed by a Creative Commons licence – details of these licences and which Creative Commons licence will apply to this Work are set out in our licence referred to above.

Other than as permitted in any relevant BMJ Author's Self Archiving Policies, I confirm this Work has not been accepted for publication elsewhere, is not being considered for publication elsewhere and does not duplicate material already published. I confirm all authors consent to publication of this Work and authorise the granting of this licence.

For peer review only

Health and wellbeing after being deployed in a major incident; how do Swedish ambulance nurses perceive their health recover process? A qualitative study.

Authors:

Karin Blomberg, Faculty of Medicine and Health, School of Health Sciences, Örebro university, Örebro, Sweden.

Karin Hugelius*, Faculty of Medicine and Health, School of Health Sciences, Örebro university, Örebro, Sweden.

* Corresponding author

Corresponding author contacts:

Karin Hugelius
E-mail: karin.hugelius@oru.se
Phone: +46 70 219 59 35

Word count: 4099

For peer review only

ABSTRACT

Objectives: To explore health problems and the recovery process after being deployed in a major incident.

Design: Qualitative, explorative design.

Setting: Ambulance services in Sweden.

Participants and methods: Semi-structured, individual two-session interviews with 15 ambulance nurses with the experience of being deployed to major incidents were conducted. Data were analysed with thematic analysis.

Results: Being deployed in major incidents was perceived as straining and led to both physical health problems and distress. To recover, the ambulance nurses strived to use strategies to distance themselves from the situation and created supportive conditions for their recovery, and if successful, the experiences led to both professional and personal growth and self-awareness. However, being deployed in major incidents without significant preparedness or experience could harm individuals and, in the worst case, end their career.

Conclusions: A successful recovery from the physical and mental exhaustion experienced after being deployed in a major incident required both individual abilities and self-care strategies as well as a supportive working environment. Supporting individual recovery strategies and following up on physical and mental well-being over time should be part of all ambulance services procedures after major incidents.

Keywords: ambulance personnel, ambulance nurses, major incident, resilience, stress, work-related stress, recovery

ARTICLE SUMMERY

Strengths and limitations of this study

- The two-session interviews used stimulated a deeper reflection and rich material to analyse.
- Information power was considered as sufficient.
- Data were analysed and confirmed by researchers with different perspectives on the topic.
- It can never be excluded that another study sample, the characteristics of the major events forming the basis of this study, or the timing of the data collection might have influenced the results.

BACKGROUND

Ambulance personnel are, daily, subject to situations that can evoke distress, but also occasional events such as major incidents, mass casualty situations or disasters that might adversely affect their well-being [1 2]. Responding to major incidents, such as large accidents, terrorist attacks or natural disasters, is a challenging experience even for experienced and trained professionals. Previous studies suggest that such experiences can have both physical and psychological health consequences among first responders [3-5]. After the 9/11 terrorist attack, musculoskeletal problems, breathing problems and undefined bodily pain were reported among the first responders [5]. Among mental health problems, post-traumatic stress disorder (PTSD) seems to be the most frequently studied

For peer review only

condition, and the risk for PTSD has been found to be higher among ambulance personnel than in the general population [3 6]. In particular, professionals in a command position during a major incident or disaster have been found to have an increased risk for mental health problems [3].

Resilience has been described as the ability to "bounce back" after a displacement [7]. It does not necessarily mean the absence of stress reactions but that the person recovers, can carry on with important parts of their life, and can sometimes to grow from these experiences [8]. Resilience should not be seen as an individual process but as a process that is highly dependent on the setting around the person, such as social network, work organizational perspectives, and cultural and community aspects [8]. Being resilient does not necessarily mean the absence of health problems or pathology but being able to cope with the situation and actively adapt in a healthy way [9]. The resilience of ambulance nurses after being deployed in major incidents is concerning from many perspectives: for the individual affected, from an employer perspective and for the broader community that relies on their services. Especially in the aftermath of a major incident, the medical resources might be stretched, and therefore, it is of extra importance that ambulance nurses are resilient and stay healthy after such events. However, the knowledge on how to promote resilience among ambulance personnel after being deployed in major incidents is still limited. Therefore, this study aimed to explore ambulance nurses' perceived health and the recovery process after being deployed in major incidents.

METHODS

Design

An explorative, qualitative study was conducted.

Study setting

The study was conducted in Sweden. In Sweden, all emergency ambulances are staffed with at least one registered nurse, most often specialised in prehospital emergency care (called ambulance nurse) and one paramedic or a second nurse. In this study, a major incident was defined as an incident that forced the health care system to heighten the level of response due to the event.

Study sample and recruiting

Nurses or specialized nurses within the ambulance services were recruited by invitations via social media, in official or private groups specifically focused on Swedish ambulance personnel (national wise). In addition, invitations were distributed through ambulance managers in health care regions where a major incident had occurred during the last five years. Inclusion criteria were having at least one year of working experience as a nurse within the ambulance services and having first hand experiences from being deployed in a major incident during the last five years. Exclusion criteria were personal or professional relationships with any of the authors and being a paramedic (not a nurse). Eligible participants who wanted to participate in the study were asked to read full study information posted on a web page and to fill in a form with their contact information. All nurses who showed interest were contacted by a researcher (KH) via phone to set up a time for the interview. A total of 15 nurses responded to the invitation and were included in the study. No one who volunteered to participate was excluded.

Data collection

For peer review only

Two-session individual interviews [10], (n = 27) were conducted face to face (n = 2) or by phone (n = 25) with 15 nurses. All the interviews were conducted by one researcher (KH) during the period of December 2018 to February 2020 using a semi-structured interview guide. The interview guide covered questions on experiences working in a major incident, preparations for such experiences, perceived health effects and recovery strategies after such experiences. The interview guide was tested in two pilot interviews, after which only minor corrections were made. The pilot interviews were therefore included in the analysis. The study participants were asked to focus on one specific major incident, even though many of the participants expressed experiences gained from several major incidents. Probing questions, for example, "What do you mean by that…" or "Can you further describe…", were used to obtain a deeper understanding of their experiences. The second interview was conducted within 14 days of the first interview and aimed to follow up on the dialogue but also to follow up on the participant's well-being. Three participants declined a second interview due to personal circumstances such as a planned vacation, which resulted in a data set of 27 interviews in total. All interviews were audio-recorded and transcribed verbatim by a professional transcriber. The COREQ checklist was used to report the study.

Analysis

An inductive thematic analysis was used to analyse the data [11]. After transcription, the whole texts were read through several times to get familiar with the data, as the researcher (KB) primarily responsible for the analysis was not involved in data collection. Thereafter, meaning units responding to the aim of the study were extracted and coded by the first author (KH). The codes were thereafter clustered into sub-themes and themes reflecting both the coded extracts and the entire data set. This clustering formed a broader understanding of patterns within the phenomenon studied. During this phase, two researchers participated (KB, KH). A final naming of the themes and sub-themes was determined during discussions between the two researchers. Finally, the results were formulated, and quotations (from n=7 participants) were selected to illustrate the findings and to increase trustworthiness.

Ethical considerations

All participants had received written and verbal study information that their participation was voluntary and that they had the right at any time to refrain from further participation without specifying any reasons. Before the first interview, informed consent was obtained. In all, this paper presents quotations from 7 study participants. Because major incidents are rare in Sweden, no information on the study participants or the exact situation behind the specific quotation was provided to protect the participants' identity. To detect signs of potential traumatization or need for professional counselling, all study participants were asked to respond to the Posttraumatic Checklist-Civilian (PCL-IV), Swedish version. A cut-off value of over 40 was an indicator for remaining stress [12]. The research group had experience and competence to refer a study participant for further support if such a need arose. The study was approved by the Swedish Ethical Review Authority (reference number2018:232).

Patient and Public Involvement

No patients were involved in this study.

RESULTS

For peer review only

All nurses who volunteered to participate in the study were included. In all, 15 ambulance nurses, participated in the 27 interviews. The interviews lasted between 2 and 89 minutes (first interviews 24 to 89 minutes, median 52 minutes; second interviews 2 to 22 minutes, median 5 minutes). Of the study participants, ten were males and five females, aged 29 to 62 years old (median age 41), with professional experience in ambulance services varying from 1 to 39 years (median 18 years). The major incidents covered in the study included traffic accidents, fires, terrorist attacks and explosions. None of the participants screened indicated remaining traumatic stress according to the PCL-IV.

Table 1 provides an overview of the main theme and sub-themes.

Table 1. Overview of the findings

**Main themes**	**Being exhausted and vulnerable**	**Getting distance from the situation**	**Establishing supportive conditions for recovery**	**Personal and professional development**
**Sub-themes**	Experiencing physical health problems	Using self-reflection	Getting the whole picture	Professional growth
	Experiencing mental health problems	Transforming from professional to personal	Creating social support	Personal growth
	Feeling vulnerable		Experiencing professional support	Failure to cope and recover

The experiences of health problems and recovery after being deployed in a major incident could be described as a process staring right after the deployment with being physically and mentally exhausted and vulnerable, continuing with a phase of striving to find strategies that provided distance from the situation and supportive conditions for recovery, followed by an incorporation of the experiences into both personal and professional development (see Figure 1).

--INSERT Figure 1 about here--

Figure 1. The process of recovery after deployment in a major incident.

Being exhausted and vulnerable

After finalizing the deployment in the major incident, all nurses described feelings of being totally exhausted, both physically and mentally. Physical health problems such as a general bodily pain, headache or dizziness were most intense during the first few days. A tiredness that was overwhelming was described; at the same time, sleeping problems could be present. The nurses described that their minds and thoughts were constantly busy processing impressions from the

For peer review only

major incident, as a kind of background music, making it hard to relax. Additionally, cognitive problems, such as having difficulties concentrating, were reported. Most mental reactions started after some time and were more intense a few days after the incident.

> "I was so tired. Extremely tired. Had some pain in my body. Needed to rest. And then…the thought went like in a loop- coming back all the time; what you saw, what you did. I kind of went through the situation all over again and again. It lasted for some day maybe. Then I could move forward and put it behind me."

The experience of being exhausted was accompanied by feelings of being vulnerable. Being faced with how the incident had caused personal tragedies and affected human people reminded them of the fragility of life and made the nurses feel vulnerable.

> "But then…. after a while. It was very hard actually. But it took three days until I could take it in: those things I've seen there in the bus. I read about the victims in a newspaper. Their families spoke about them, of course. Then I felt….it was tough…I became sad. "

The nurses sometimes put themselves in the position of the injured or family members, as if the incident had affected themselves or their own lives. That increased feelings of being sad and powerless. Sometimes, existential thoughts occurred, such as questioning the meaning of life or the meaning of the incident that had occurred.

Getting distance from the situation

After having overcome the exhaustion that was experienced in the first phase, the nurses strived to get distance from the major incident. For some, this phase started only a few hours after the incident, whereas for others, it could be some days after the incident. Being alone, taking one's time to reflect on the actual incident as well as one's own emotions and thoughts, and finding one's own "inner place" were essential and contributed to decreased physical problems as well as decreased distress. Another strategy used to get distance from the situation was to return as soon as possible to everyday life and routines, both personal and professional. Such activities and contexts included everyday practices such as eating together and watching TV with colleagues, including those they had worked with during the major incident, as a method of "normalization". Using humour, for example, giggling together about other things that had occurred during the incident, gave them distance from the experiences.

> ".”

> "For me, it was taking a shower, getting the ambulance uniform off, eat and walk. We laughed a lot during the dinner. We ordered food, and sat around the table and in fact, it was a kind of rambunctious atmosphere. I think it a kind of art, to be able to laugh and relax, despite what has happened. It releases the stress…."

Several of the nurses described activities that could be understood as rituals to handle the transition from being a professional during the major incident to becoming a private person. An example of such rituals was taking a bath or shower to cleanse oneself of the experienced situation.

For peer review only

> "They had made sandwiches and so, but I could not eat. My body was on high alert so to say, all I wanted was to take a shower, to wash the incident off myself, to clean myself from it in some way…. I did not want to talk about it actually…. "

Most of the nurses claimed that they preferred to stay with their colleagues and to keep on working after the major incident, even if offered to go home immediately. Staying at work also contributed to normalization and de-dramatized the situation.

Establishing supportive conditions for recovery

A significant part of the individual responsibility of each nurse was to create good conditions for their own recovery. However, the preferred conditions varied widely. A common understanding was that stress reactions were to be seen as normal but still required quite some time to recover from. Participants with less professional experience described how they were unprepared for the time and energy required to recover from working during major incidents. Creating their "own story" of the incident and their efforts sometimes required information from other perspectives or persons involved. One example of such information needed could be information about the victims' outcome.

> "Well, for me, I had the whole picture, from alarm to the end so to say, but many of our colleagues had only arrived and picked up a patient they took to the hospital. And they needed to get information on what had happened before they arrived….and I needed information on what had happened to the patient during the transport. So, we spend some time to talk about these things, to clear the scenario out for everyone"

If there were fatalities, some nurses conducted private rituals, such as lighting a candle for the victims. Such rituals could serve as an endpoint of the major incident. Creating possibilities of informal sharing of experiences with others who understood the context, such as colleagues or first-line managers, was a crucial part of the supportive environment. Such sharing also contributed to a feeling of being part of the team and getting confirmation of one's efforts during the major incident.

> "I think that it is very important with the colleagues, especially those who have long experience, who have seen most things and you can say anything- they can bear to hear it. Because they have seen it themselves, some time. I cannot tell anyone else; they will not understand, and they will distance themselves. I know that my colleagues understand when I have to make a joke about something horrible, because there is no other way to cope with it…That is a respect for the human being as I don't recognize from any other profession."

Almost all nurses reflected on individual self-reflection and informal social support compared with organized psychosocial support, such as after-action reviews or group sessions including psychological debriefing. Many of the nurses considered such organized sessions not to be supportive of their recovery, and for some, they had an opposite effect than intended. Additionally, individuals who did not feel like talking or being around colleagues at all had to be respected. A general caring attitude from colleagues and first-line managers was important to create trust that if someone needed professional support, such support would be immediately provided. Because most mental reactions occurred after some time following the incident, many participants meant that the concern and care from colleagues and managers also had to be present after some time and that offers of professional support were more valuable after some time than during the acute phase.

For peer review only - http://bmjopen.bmj.com/site/about/guidelines.xhtml

Personal and professional development

The experiences gained both from the major incident itself but also from reactions and the following recovery process needed to be integrated into the personal and professional identity and could then contribute to professional and sometimes personal growth. Some nurses stated that this process of incorporating the major event as a part of their overall experiences was a skill that could be learned over years within the emergency medical services. To facilitate this transformation, continuing to take part in ordinary work, accepting the distress and relying on the individual recovery process were supportive. Senior colleagues and managers could play an important role in this process by confirming the occurrence of distress and sharing their experiences of similar recovery processes. At the same time as it was perceived as important to finalize and "store" their experiences, the nurses expressed worries about what impact such strong experiences would have on them as both professionals and people in a longer perspective.

> "My fiancé came home and just held me. We didn't talk, it wasn't necessary. He just held me. I thought that I had to quit this job. But at the same time; it is a part of me, who I am. I want to be in situations like this. I want to help. I'm an ambulance nurse, in my person. And so is he. It's part of our lives, that we understand each other's needs in situations like this. I was pregnant that day. He was the only person who knew. I was so worried for the future…."

Over time, new perspectives on life, on being a human and on the role of the emergency medical services developed, mostly adding wisdom and being more resilient, as both a person and as a nurse. Most often, this process was positive, leading to an increased self-awareness and ability to manage and endure severe situations.

> "Some new people that come to us think that it is a tough environment within the ambulance services, that we are hard as stone. I say it is the opposite. We witness a lot, we meet people in all kinds of situations, when they are at most vulnerable, when they are between life or death, when they don't want to live any more, when they are severely beaten…and that could happen to us all. Everyone. Life is not always clean and tidy, not always fair. You realize that after some years. We can laugh about it, together, about things that are not really fun for anyone else. Undramatized it. We live today, but you never know about tomorrow… You learn that within the ambulance services. I think it is good. You need that reminder sometimes. And I live a richer life thanks to that."

However, sometimes, the burden of the experiences from the major event was too hard to endure, and the recovery process did not end with a positive development. In such situations, the nurses felt overwhelmed and unsuccessful, which led to persistent physical or mental health problems and in some cases, an ending of their career.

> "My colleague, she was incident commander in an accident where two children died. She left the ambulance services after that. She just cried as soon as she came to the work. It was very hard to see her, actually. She was a very competent ambulance nurse, but this crushed her. I tried, we tried all of us. But she took it very personally, she withered away…"

For peer review only

The risk for such outcomes was experienced as higher if the nurse was unexperienced and lacked proper formal or mental preparedness for being faced with the complexity and demands of a major incident.

DISCUSSION

Being deployed as an ambulance nurse in a major incident led physical- and psychological health problems and feelings of being exhausted and vulnerable. To recover, the ambulance nurses took responsibility and strived to find strategies to gain distance from the situation and to create supportive conditions. If successful, the recovery process led to professional and personal growth. However, being deployed in major incidents without significant preparedness or experience could harm individuals and, in the worst case, end their career.

In this study, no serious physical injuries were reported, but despite that, many participants described physical health problems and exhaustion. Although physical well-being has been found to be an important mediator for psychological wellbeing [8], the physical status of ambulance nurses is important when discussing recovery after working in stressful situations.

The nurses demonstrated a high level of awareness of the occurrence of stress and the risks for being traumatized but also self-knowledge and strategies to manage distress. At the same time, several expressed worries about colleagues and sometimes fear of being exposed to situations that might overload their capacity to recover. A realistic appraisal and active decision-making on how to cope with the situation can contribute to resilience after stressful events [7]. In this study, the participants described different activities that can be seen as "rituals", striving for balance concerning professional boundaries between themselves as a person and being an ambulance personnel. It seems that these "rituals" also mediated a transition from "being in the major incident" to "being after the incident" and moving to a more ordinary everyday life. In the theoretical framework of transition [13], this process has been described as a passage from one phase, condition or status to another or as a period between relative stable phases. The transition process is characterised by disconnecting with previous contexts and believes, moving through a period of unfamiliarity and finally the replacement of an existing set of expectations with new ones[14]. The transition, as well as resilience, is depended not only on the individual, but on the social contexts. For a successful transition process, the person needs to have awareness of both their situation and the transition process, and the level of engagement is essential. To develop skills for transition, both supportive measures such as mentorship has been suggested, but also, being exposed to regular transitioning produces prepare individuals to cope with challenges[14]. Future studies are suggested to explore the relationship between transition and resilience among health professionals exposed to potentially traumatic events.

The participants in this study expressed a strong commitment and convention that they were accountable themselves for creating their own recovery process. Being aware of one's own responsibility for one's well-being and using potential traumatic events and severe stress as a foundation to learn and grow from [8] can be applied to the results of this study. All transitions or changes also require time [13], which was strongly emphasized by the study participants who expressed that they sometimes were surprised that their own recovery took longer than expected.

Self-care has been found to be a key to protect against negative health impacts [15]. The study also confirmed that ambulance nurses need to acquire a unique set of personal qualities to promote self-

care and self-resilience in their everyday work [16]. A question raised was whether the competence to develop self-care strategies and use experiences to grow is something that can developed over time or whether it is personal attribute needed to be comfortable in a profession such as being a nurse within the ambulance services. Is there a self-selection component that can support resilience within the ambulance services, or is it a result of a professional development process over time?

To mitigate the negative health effects and promote physical and mental wellbeing requires an awareness of the demands as well as the recovery process. Prevention of work-related traumatic stress should rely on a sound psychosocial work environment, systematic training of employees, social support from colleagues and managers, and proper follow-up of employees after a critical event [17]. Well-functioning supportive structures and working environment have been suggested to compensate for short professional experiences [15] and might therefore be of extra importance for less experienced ambulance personnel. Social support seems particularly powerful to reduce negative outcomes of stress [15 18 19]. In line with evidence, organized sessions with the aim to process emotions in the immediate aftermath of the potentially traumatic event, such as debriefings or group counselling, were not experiences that contributed to recovery or resilience. In accordancy with previous evidence, such interventions should therefore not be part of the organizational support systems for ambulance personnel after being deployed to major incidents [20 21]. Instead, this study emphasizes the employer's responsibility to understand the recovery process, to enable time and space for the recovery, and to systematically and with empathy follow up on both physical and mental health, both in the acute phase and over time. Ensuring that ambulance personnel are "fit for duty", in terms of being physically and mentally ready to respond to challenging situations and also being prepared to recover after such situations, after being deployed in major incidents should therefore be a shared responsibility between the individual professional and the employer as a natural component in a resilient ambulance service both in the every day services and in major incidents. Also, further scientific interest in what demands that are required to respond to such incidents and how such qualities develop is needed.

Limitations

As in all qualitative research studies, it can never be excluded that another study sample, the characteristics of the major events forming the basis of this study, or the timing of the data collection might have influenced the results. However, the study sample size was considered to have information power [22], influenced by recruiting study participants with quite recent experiences, high-quality dialogue during the interviews, the two-session interviews and the use of a systematic analysis methodology. None of the included nurses reported worrisome levels of traumatic stress according to the screening. However, it may be that nurses who have left the ambulance services following a potentially traumatic experiences were less likely to be included in this study, given the recruitment procedure, and this must be considered as a limitation.

The two-session interview design, chosen to ensure that all data were collected and to ensure the well-being of the study participants. The second interviews were in general shorter than the first ones and contributed to rich data by confirming the experiences that were expressed in the first interviews, but also to verifying the researchers´ interpretations of the first interview statements. No new topics were expressed in the second interviews. All data were analysed and confirmed by researchers with different perspectives on the topic, including an extensive preunderstanding of the

For peer review only

ambulance services, major incidents and caring in emotionally challenging situations, which was found to contribute to the understanding of the data.

CONCLUSION

The ambulance nurses demonstrated a high level of awareness of the occurrence of stress and individual responsibility to find strategies to recover. A successful recovery from the physical and mental exhaustion experienced after being deployed in a major incident required both individual abilities and self-care strategies as well as a supportive working environment. Organized sessions such as debriefings or group counselling did not contribute to recovery and should therefore not be part of the routine organizational support systems. Instead, supporting individual recovery strategies and following up on physical and mental well-being both in the acute phase and over time should be part of all ambulance services procedures after major incidents to promote individual and organizational resilience. Since the resilience of ambulance personnel is essential for a well-functioning emergency services, further research on how to promote such resilience is strongly needed.

List of abbreviations

Post-traumatic stress disorder (PTSD)

Posttraumatic Checklist-Civilian (PCL)

Funding

This research received no specific grant from any funding agency in the public, commercial or not-for-profit sectors.

DECLARATIONS

Ethics approval and consent to participate
The study was reviewed and approved by the Swedish Ethical Review Authority (ref id 2018:232).

Availability of data and materials
The data analysed during the current study are not publicly available due to Swedish laws on research ethics and the ethical approval provided by the Swedish Ethical Review Authority.

Competing interests
The authors declare that they have no competing interests.

Author contributions
KB: Conceptualization, analysis, writing of the manuscript, KH: Conceptualization, data collection, writing of the manuscript. Both authors read and approved the final manuscript.

Acknowledgement
The authors would like to thank Dr Samuel Edelbring for his valuable contribution to the initial design and planning of the data collection for this study.

For peer review only

References

1. Halpern J, Maunder RG, Schwartz B, et al. Identifying, Describing and Expressing Emotions After Critical Incidents in Paramedics. *Journal of Traumatic Stress* 2012;25:111-14.
2. Lawn S, Roberts L, Willis E, et al. The effects of emergency medical service work on the psychological, physical, and social well-being of ambulance personnel: a systematic review of qualitative research. *BMC Psychiatry* 2020;20(1):348.
3. Berger W, Coutinho ES, Figueira I, et al. Rescuers at risk: a systematic review and meta-regression analysis of the worldwide current prevalence and correlates of PTSD in resuce workers. *Social Psychiatry and Psychiatric Epidemiology* 2012;47(6):1001-11.
4. Skogstad L, Heir T, Hauff E, et al. Post traumati stress among rescue workers after terror attacks in Norway. *Occupational and Enivornmental Medicine* 2016;66(7):528-35.
5. Smith EC, Holmes L, Burkle FM. The Physical and Mental Health Challenges Experienced by 9/11 First Responders and Recovery Workers: A Review of the Literature. *Prehospital and Disaster Medicine* 2019 Dec;34(6):625-631.
6. Petrie K, Milligan-Saville J, Gayed A, et al. Prevalence of PTSD and common mental disorders amongst ambulance personnel: a systematic review and meta-analysis. *Social Psychiatry and Psychiatric Epidemiology* 2018;53(9):897-909.
7. Southwick SM, Bonanno GA, Masten AA, et al. Resilience definitions, theory, and challenges: interdisciplinary perspectives. *European Journal of Psychotraumatology* 2014;1(5):25338.
8. Southwick SM, Charney DS. Resilience. The Science of Mastering Life´s Greatest Challanges. Second ed.New York: Cambridge University Press 2018.
9. Shakespeare-Finch J, Daley E. Workplace belongingness, distress, and resilience in emergency service workers. *Psychological Trauma: Theory, Research, Practice, and Policy* 2017;9(1):32-35.
10. Read BL. Serial Interviews: When and Why to Talk to Someone More Than Once. *International Journal of Qualitative Methods* 2018; 17.(1).
11. Braun V, Clarke, V. Using thematic analysis in psychology. *Qualitative Research in Psychology* 2006;3(2):77-101.
12. Arnberg F, Michel PO, Johannesson KB. Properties of Swedish Posttraumatic Stress Measures after a Disaster. *Journal of Anxiety Disorders* 2014;28(4):402-09.
13. Meleis AI, Sawyer LM, Im EO, et al. Experiencing transitions: an emerging middle-range theory. *Advancec in Nursing Science* 2000;23(1):12-28.
14. Al-Yateem N, Docherty, C. Transition: A concept of significance to nursing and health care professionals. *Journal of Nursing Education and Practice*. 2015;5(5):35-40
15. Smith E, Walker T, Burkle FM. Lessons in Post-Disaster Self-Care From 9/11 Paramedics and Emergency Medical Technicians. *Prehospital and Disaster Medicine* 2019;34(3):335-39.
16. Clompus SR, Albarran JW. Exploring the nature of resilience in paramedic practice: A psycho-social study. *International Emergency Nursing* 2016;28:1-7.
17. Skogstad L, Skorstad, M., Lie, A., Conradi, H.S., Heir, T., Weisaeth, L. Work-related post- traumatic stress disorder. *Occupational Medicine* 2013;63(3):175-82.
18. Behnke A, Rojas R, Karrasch S, et al. Deconstructing Traumatic Mission Experiences: Identifying Critical Incidents and Their Relevance for the Mental and Physical Health Among Emergency Medical Service Personnel. *Frontiers in Psychologt* 2019;10:2305.
19. Arnberg F, Hultman CM, Michel M, et al. Social Support Moderates Posttraumatic Stress and General Distress After Disaster. *Journal of Traumatic Stress* 2012;25(6):721-27.
20. Rose SC, Bisson J, Churchill R, et al. Pshycological debriefing for preventing post traumatic stress disorder (PTSD) (Review). Cochrane Database Syst Rev: The Cochrane Library, 2006.
21. International Society for Traumatic Stress Studies. Posttraumatic Stress Disorder Prevention and Treatment Guidelines. Methodology and Recommendations. 2019. Avalibe at: https://istss.org/getattachment/Treating-Trauma/New-ISTSS-Prevention-and-Treatment-Guidelines/ISTSS_PreventionTreatmentGuidelines_FNL.pdf.aspx. Accessed: January 09, 2023.

For peer review only

22. Malterud K, Siersma VD, Guassora AD. Sample Size in Qualitative Interview Studies: Guided by Information Power. *Qualitative Health Reseach* 2016;26(13):1753-60.

For peer review only

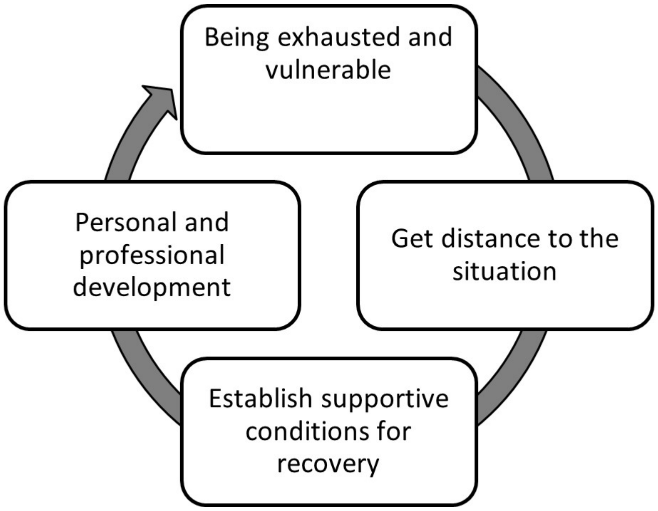

Figure 1. The process of recovery after deployment in a major incident.

107x54mm (330 x 330 DPI)

COREQ (COnsolidated criteria for REporting Qualitative research) Checklist

A checklist of items that should be included in reports of qualitative research. You must report the page number in your manuscript where you consider each of the items listed in this checklist. If you have not included this information, either revise your manuscript accordingly before submitting or note N/A.

Topic	Item No.	Guide Questions/Description	Reported on Page No.
**Domain 1: Research team and reflexivity**			
Personal characteristics			
Interviewer/facilitator	1	Which author/s conducted the interview or focus group?	
Credentials	2	What were the researcher's credentials? E.g. PhD, MD	
Occupation	3	What was their occupation at the time of the study?	
Gender	4	Was the researcher male or female?	
Experience and training	5	What experience or training did the researcher have?	
Relationship with participants			
Relationship established	6	Was a relationship established prior to study commencement?	
Participant knowledge of the interviewer	7	What did the participants know about the researcher? e.g. personal goals, reasons for doing the research	
Interviewer characteristics	8	What characteristics were reported about the inter viewer/facilitator? e.g. Bias, assumptions, reasons and interests in the research topic	
**Domain 2: Study design**			
Theoretical framework			
Methodological orientation and Theory	9	What methodological orientation was stated to underpin the study? e.g. grounded theory, discourse analysis, ethnography, phenomenology, content analysis	
Participant selection			
Sampling	10	How were participants selected? e.g. purposive, convenience, consecutive, snowball	
Method of approach	11	How were participants approached? e.g. face-to-face, telephone, mail, email	
Sample size	12	How many participants were in the study?	
Non-participation	13	How many people refused to participate or dropped out? Reasons?	
Setting			
Setting of data collection	14	Where was the data collected? e.g. home, clinic, workplace	
Presence of non-participants	15	Was anyone else present besides the participants and researchers?	
Description of sample	16	What are the important characteristics of the sample? e.g. demographic data, date	
Data collection			
Interview guide	17	Were questions, prompts, guides provided by the authors? Was it pilot tested?	
Repeat interviews	18	Were repeat inter views carried out? If yes, how many?	
Audio/visual recording	19	Did the research use audio or visual recording to collect the data?	
Field notes	20	Were field notes made during and/or after the inter view or focus group?	
Duration	21	What was the duration of the inter views or focus group?	
Data saturation	22	Was data saturation discussed?	
Transcripts returned	23	Were transcripts returned to participants for comment and/or	

For peer review only

Topic	Item No.	Guide Questions/Description	Reported on Page No.
		correction?	
**Domain 3: analysis and findings**			
Data analysis			
Number of data coders	24	How many data coders coded the data?	
Description of the coding tree	25	Did authors provide a description of the coding tree?	
Derivation of themes	26	Were themes identified in advance or derived from the data?	
Software	27	What software, if applicable, was used to manage the data?	
Participant checking	28	Did participants provide feedback on the findings?	
Reporting			
Quotations presented	29	Were participant quotations presented to illustrate the themes/findings? Was each quotation identified? e.g. participant number	
Data and findings consistent	30	Was there consistency between the data presented and the findings?	
Clarity of major themes	31	Were major themes clearly presented in the findings?	
Clarity of minor themes	32	Is there a description of diverse cases or discussion of minor themes?	

Developed from: Tong A, Sainsbury P, Craig J. Consolidated criteria for reporting qualitative research (COREQ): a 32-item checklist for interviews and focus groups. *International Journal for Quality in Health Care*. 2007. Volume 19, Number 6: pp. 349 – 357

Once you have completed this checklist, please save a copy and upload it as part of your submission. DO NOT include this checklist as part of the main manuscript document. It must be uploaded as a separate file.

For peer review only

BMJ Open

Health and wellbeing after being deployed in a major incident; how do Swedish ambulance nurses perceive their health recover process? A qualitative study.

Journal:	*BMJ Open*
Manuscript ID	bmjopen-2023-071848.R2
Article Type:	Original research
Date Submitted by the Author:	01-May-2023
Complete List of Authors:	Blomberg, Karin; Örebro University, Faculty of Medicine and Health<br>Hugelius, Karin; Örebro University, Faculty of Medicine and Health
**Primary Subject Heading**:	Emergency medicine
Secondary Subject Heading:	Nursing, Qualitative research
Keywords:	ACCIDENT & EMERGENCY MEDICINE, Health & safety < HEALTH SERVICES ADMINISTRATION & MANAGEMENT, QUALITATIVE RESEARCH

SCHOLARONE™
Manuscripts

I, the Submitting Author has the right to grant and does grant on behalf of all authors of the Work (as defined in the below author licence), an exclusive licence and/or a non-exclusive licence for contributions from authors who are: i) UK Crown employees; ii) where BMJ has agreed a CC-BY licence shall apply, and/or iii) in accordance with the terms applicable for US Federal Government officers or employees acting as part of their official duties; on a worldwide, perpetual, irrevocable, royalty-free basis to BMJ Publishing Group Ltd ("BMJ") its licensees and where the relevant Journal is co-owned by BMJ to the co-owners of the Journal, to publish the Work in this journal and any other BMJ products and to exploit all rights, as set out in our licence.

The Submitting Author accepts and understands that any supply made under these terms is made by BMJ to the Submitting Author unless you are acting as an employee on behalf of your employer or a postgraduate student of an affiliated institution which is paying any applicable article publishing charge ("APC") for Open Access articles. Where the Submitting Author wishes to make the Work available on an Open Access basis (and intends to pay the relevant APC), the terms of reuse of such Open Access shall be governed by a Creative Commons licence – details of these licences and which Creative Commons licence will apply to this Work are set out in our licence referred to above.

Other than as permitted in any relevant BMJ Author's Self Archiving Policies, I confirm this Work has not been accepted for publication elsewhere, is not being considered for publication elsewhere and does not duplicate material already published. I confirm all authors consent to publication of this Work and authorise the granting of this licence.

For peer review only

Health and wellbeing after being deployed in a major incident; how do Swedish ambulance nurses perceive their health recover process? A qualitative study.

Authors:

Karin Blomberg, Faculty of Medicine and Health, School of Health Sciences, Örebro university, Örebro, Sweden.

Karin Hugelius*, Faculty of Medicine and Health, School of Health Sciences, Örebro university, Örebro, Sweden.

* Corresponding author

Corresponding author contacts:

Karin Hugelius
E-mail: karin.hugelius@oru.se
Phone: +46 70 219 59 35

Word count: 4380

For peer review only

ABSTRACT

Objectives: To explore health problems and the recovery process after being deployed in a major incident.

Design: Qualitative, explorative design.

Setting: Ambulance services in Sweden.

Participants and methods: Semi-structured, individual two-session interviews with 15 ambulance nurses with the experience of being deployed to major incidents were conducted. Data were analysed with thematic analysis.

Results: Being deployed in major incidents was perceived to be straining and led to both physical health problems and distress. To recover, the ambulance nurses strived to use strategies to distance themselves from the situation and created supportive conditions for their recovery, and if successful, the experiences led to both professional and personal growth and self-awareness. However, being deployed in major incidents without significant preparedness or experience could harm individuals and, in the worst case, end their career.

Conclusions: A successful recovery from the physical and mental exhaustion experienced after being deployed in a major incident required both individual abilities and self-care strategies as well as a supportive working environment. Supporting individual recovery strategies and following up on physical and mental well-being over time should be part of all ambulance services procedures after major incidents.

Keywords: ambulance personnel, ambulance nurses, major incident, resilience, stress, work-related stress, recovery

ARTICLE SUMMERY

Strengths and limitations of this study

- The two-session interviews used stimulated a deeper reflection and rich material to analyse.
- Information power was considered as sufficient.
- Data were analysed and confirmed by researchers with different perspectives on the topic.
- It can never be excluded that another study sample, the characteristics of the major events forming the basis of this study, or the timing of the data collection might have influenced the results.

BACKGROUND

Ambulance personnel are, daily, subject to situations that can evoke distress, but also occasional events such as major incidents, mass casualty situations or disasters that might adversely affect their well-being [1 2]. Responding to major incidents, such as large accidents, terrorist attacks or natural disasters, is a challenging experience even for experienced and trained professionals. Previous studies suggest that such experiences can have both physical and psychological health consequences among first responders [3-5]. After the 9/11 terrorist attack, musculoskeletal problems, breathing problems and undefined bodily pain were reported among the first responders [5]. Among mental health problems, post-traumatic stress disorder (PTSD) seems to be the most frequently studied

For peer review only

condition, and the risk for PTSD has been found to be higher among ambulance personnel than in the general population [3 6]. In particular, professionals in a command position during a major incident or disaster have been found to have an increased risk for mental health problems [3].

Resilience has been described as the ability to “bounce back” after a displacement [7]. It does not necessarily mean the absence of stress reactions but that the person recoversand can sometimes to grow from these experiences [8]. Resilience should be seen as a process that is highly dependent on the setting around the person, such as social network, work organizational perspectives, and cultural and community aspects [8]. Being resilient does not necessarily mean the absence of health problems or pathology but being able to cope with the situation and actively adapt in a healthy way [9]. The resilience of ambulance nurses after being deployed in major incidents is concerning for the individual affected, from an employer perspective and for the broader community that relies on their services. Especially in the aftermath of a major incident, the medical resources might be stretched, and therefore, it is of extra importance that ambulance nurses are resilient and stay healthy after such events. However, the knowledge on how to promote resilience among ambulance personnel after being deployed in major incidents is still limited. Therefore, this study aimed to explore ambulance nurses’ perceived health and the recovery process after being deployed in major incidents.

METHODS

Design

An explorative, qualitative study was conducted.

Study setting

The study was conducted in Sweden. In Sweden, all emergency ambulances are staffed with at least one registered nurse, most often specialised in prehospital emergency care (called ambulance nurse) and one paramedic or a second nurse. In this study, a major incident was defined as an incident that forced the health care system to heighten the level of response due to the event.

Study sample and recruiting

The data used in this study was gathered simultaneously as data for another study, using the same study sample[10]. Nurses or specialized nurses within the ambulance services were recruited by invitations via social media, in official or private groups specifically focused on Swedish ambulance personnel (national wide). In addition, invitations were distributed through ambulance managers in health care regions where a major incident had occurred recently.. Inclusion criteria were having at least one year of working experience as a nurse within the ambulance services and having first hand experiences from being deployed in a major incident during the last five years. Exclusion criteria were personal or professional relationships with any of the authors and being a paramedic (not a nurse). Eligible participants who wanted to participate in the study were asked to read full study information posted on a web page and to fill in a form with their contact information. All nurses who showed interest were contacted by a researcher (KH) to set up a time for the interview. A total of 15 nurses responded to the invitation and were included in the study. No one who volunteered to participate was excluded.

Data collection

For peer review only - http://bmjopen.bmj.com/site/about/guidelines.xhtml

Two-session individual interviews [11], (n = 27) were conducted face to face (n = 2) or by phone (n = 25) (depending on geographical distance and the choice of the study person)with 15 nurses. All the interviews were conducted by one researcher (KH) during the period of December 2018 to February 2020 using a semi-structured interview guide. The interview guide covered questions on experiences working in a major incident, preparations for such experiences, perceived health effects and recovery strategies after such experiences. The interview guide was tested in two pilot interviews, after which only minor corrections were made. The pilot interviews were therefore included in the analysis. The study participants were asked to focus on one specific major incident, even though many of the participants expressed experiences gained from several major incidents. Probing questions, for example, "What do you mean by that…" or "Can you further describe…", were used to obtain a deeper understanding of their experiences. The second interview was conducted within 14 days of the first interview and aimed to follow up on the dialogue but also to follow up on the participant's well-being. Three participants declined a second interview due to personal circumstances such as a planned vacation, which resulted in a data set of 27 interviews in total. All interviews were audio-recorded and transcribed verbatim by a professional transcriber. The COREQ checklist was used to report the study.

Analysis

An inductive thematic analysis was used to analyse the data [12]. After transcription, the whole texts were read through several times to get familiar with the data, as the researcher (KB) primarily responsible for the analysis was not involved in data collection. Thereafter, meaning units responding to the aim of the study were extracted and coded by the first author (KH). The codes were thereafter clustered into sub-themes and themes reflecting both the coded extracts and the entire data set. This clustering formed a broader understanding of patterns within the phenomenon studied. During this phase, two researchers participated (KB, KH). A final naming of the themes and sub-themes was determined during discussions between the two researchers. Finally, the results were formulated, and quotations (from n=7 participants) were selected to illustrate the findings and to increase trustworthiness.

Ethical considerations

All participants had received written and verbal study information that their participation was voluntary and that they had the right at any time to refrain from further participation without specifying any reasons. Before the first interview, informed consent was obtained. In all, this paper presents quotations from 7 study participants. Because major incidents are rare in Sweden, no information on the study participants or the exact situation behind the specific quotation was provided to protect the participants' identity. To detect signs of potential traumatization or need for professional counselling, all study participants were asked to respond to the Posttraumatic Checklist-Civilian (PCL-IV), Swedish version. A cut-off value of over 40 was an indicator for remaining stress [13]. The study was approved by the Swedish Ethical Review Authority (reference number2018:232).

Patient and Public Involvement

No patients were involved in this study.

RESULTS

For peer review only

All nurses who volunteered to participate in the study were included. In all, 15 ambulance nurses, participated in the 27 interviews. The interviews lasted between 2 and 89 minutes (first interviews 24 to 89 minutes, median 52 minutes; second interviews 2 to 22 minutes, median 5 minutes). Of the study participants, ten were males and five females, aged 29 to 62 years old (median age 41), with professional experience in ambulance services varying from 1 to 39 years (median 18 years). The major incidents covered in the study included traffic accidents, fires, terrorist attacks and explosions. None of the participants screened indicated remaining traumatic stress according to the PCL-IV. Table 1 provides an overview of the main theme and sub-themes.

Table 1. Overview of the findings

**Main themes**	**Being exhausted and vulnerable**	**Getting distance from the situation**	**Establishing supportive conditions for recovery**	**Personal and professional development**
**Sub-themes**	Experiencing physical health problems	Using self-reflection	Getting the whole picture	Professional growth
	Experiencing mental health problems	Transforming from professional to personal	Creating social support	Personal growth
	Feeling vulnerable		Experiencing professional support	Failure to cope and recover

The experiences of health problems and recovery after being deployed in a major incident could be described as a process staring right after the deployment with being physically and mentally exhausted and vulnerable, continuing with a phase of striving to find strategies that provided distance from the situation and supportive conditions for recovery, followed by an incorporation of the experiences into both personal and professional development (see Figure 1).

--INSERT Figure 1 about here--

Figure 1. The process of recovery after deployment in a major incident.

Being exhausted and vulnerable

After finalizing the deployment in the major incident, all nurses described feelings of being totally exhausted, both physically and mentally. Physical health problems such as a general bodily pain, headache or dizziness were most intense during the first few days. A tiredness that was overwhelming was described; at the same time, sleeping problems could be present. The nurses described that their minds and thoughts were constantly busy processing impressions from the major incident, as a kind of background music, making it hard to relax. Additionally, cognitive

For peer review only

problems, such as having difficulties concentrating, were reported. Most mental reactions started after some time and were more intense a few days after the incident.

> "I was so tired. Extremely tired. Had some pain in my body. Needed to rest. And then…the thought went like in a loop- coming back all the time; what you saw, what you did. I kind of went through the situation all over again and again. It lasted for some day maybe. Then I could move forward and put it behind me."

The experience of being exhausted was accompanied by feelings of being vulnerable. Being faced with how the incident had caused personal tragedies and affected human people reminded them of the fragility of life and made the nurses feel vulnerable.

> "But then…. after a while. It was very hard actually. But it took three days until I could take it in: those things I've seen there in the bus. I read about the victims in a newspaper. Their families spoke about them, of course. Then I felt….it was tough…I became sad. "

The nurses sometimes put themselves in the position of the injured or family members, as if the incident had affected themselves or their own lives. That increased feelings of being sad and powerless. Sometimes, existential thoughts occurred, such as questioning the meaning of life or the meaning of the incident that had occurred.

Getting distance from the situation

After having overcome the exhaustion, the nurses strived to get distance from the major incident. For some, this phase started only a few hours after the incident, whereas for others, it could be some days after the incident. Being alone, taking one's time to reflect on the actual incident as well as one's own emotions and thoughts, and finding one's own "inner place" were essential and contributed to recovery. Another strategy used was to return as soon as possible to everyday life and routines, both personal and professional. Such activities and contexts included everyday practices such as eating together and watching TV with colleagues, including those they had worked with during the major incident, as a method of "normalization". Using humour, for example, giggling together about other things that had occurred during the incident, gave them distance from the experiences.

> "For me, it was taking a shower, getting the ambulance uniform off, eat and walk. We laughed a lot during the dinner. We ordered food, and sat around the table and in fact, it was a kind of rambunctious atmosphere. I think it a kind of art, to be able to laugh and relax, despite what has happened. It releases the stress…."

Several of the nurses described activities that could be understood as rituals to handle the transition from being a professional during the major incident to becoming a private person. An example of such rituals was taking a bath or shower to cleanse oneself of the experienced situation.

> "They had made sandwiches and so, but I could not eat. My body was on high alert so to say, all I wanted was to take a shower, to wash the incident off myself, to clean myself from it in some way…. I did not want to talk about it actually…. "

For peer review only

Most of the nurses claimed that they preferred to stay with their colleagues and to keep on working after the major incident, even if offered to go home. Staying at work contributed to normalization and de-dramatized the situation.

Establishing supportive conditions for recovery

A significant part of the individual responsibility was to create good conditions for their own recovery. However, the preferred conditions varied widely. Participants with less professional experience described how they were unprepared for the time and energy required to recover. Creating their "own story" of the incident and their efforts sometimes required information from other perspectives or persons involved. One example of such information needed could be information about the victims' outcome.

> "Well, for me, I had the whole picture, from alarm to the end so to say, but many of our colleagues had only arrived and picked up a patient they took to the hospital. And they needed to get information on what had happened before they arrived….and I needed information on what had happened to the patient during the transport. So, we spend some time to talk about these things, to clear the scenario out for everyone"

If there were fatalities, some nurses conducted private rituals, such as lighting a candle for the victims. Such rituals could serve as an endpoint of the major incident. Creating possibilities of informal sharing of experiences with others who understood the context, such as colleagues or first-line managers, was a crucial part of the supportive environment. Such sharing also contributed to a feeling of being part of the team and getting confirmation of one's efforts during the major incident.

> "I think that it is very important with the colleagues, especially those who have long experience, who have seen most things and you can say anything- they can bear to hear it. Because they have seen it themselves, some time. I cannot tell anyone else; they will not understand, and they will distance themselves. I know that my colleagues understand when I have to make a joke about something horrible, because there is no other way to cope with it…That is a respect for the human being as I don't recognize from any other profession."

Almost all nurses reflected on individual self-reflection and informal social support compared with organized psychosocial support, such as after-action reviews or group sessions including psychological debriefing. Many of the nurses considered such organized sessions not to be supportive of their recovery, and for some, they had an opposite effect than intended. Additionally, individuals who did not feel like talking or being around colleagues at all had to be respected. A general caring attitude from colleagues and first-line managers was important to create trust that if someone needed professional support, such support would be immediately provided. Because most mental reactions occurred after some time following the incident, many participants meant that the concern and care from colleagues and managers also had to be present after some time and that offers of professional support were more valuable after some time than during the acute phase.

Personal and professional development

The experiences gained both from the major incident itself but also from reactions and the following recovery process needed to be integrated into the personal and professional identity and could then contribute to professional and sometimes personal growth. Some nurses stated that this process of

For peer review only

incorporating the major event as a part of their overall experiences was a skill that could be learned over years within the emergency medical services. To facilitate this transformation, continuing to take part in ordinary work, accepting the distress and relying on the individual recovery process were supportive. Senior colleagues and managers could play an important role in this process by confirming the occurrence of distress and sharing their experiences of similar recovery processes. At the same time as it was perceived as important to finalize and "store" their experiences, the nurses expressed worries about what impact such strong experiences would have on them as both professionals and people in a longer perspective.

> "My fiancé came home and just held me. We didn't talk, it wasn't necessary. He just held me. I thought that I had to quit this job. But at the same time; it is a part of me, who I am. I want to be in situations like this. I want to help. I'm an ambulance nurse, in my person. And so is he. It's part of our lives, that we understand each other's needs in situations like this. I was pregnant that day. He was the only person who knew. I was so worried for the future…."

Over time, new perspectives on life, on being a human and on the role of the emergency medical services developed, mostly adding wisdom and being more resilient, as both a person and as a nurse. Most often, this process was positive, leading to an increased self-awareness and ability to manage and endure severe situations.

> "Some new people that come to us think that it is a tough environment within the ambulance services, that we are hard as stone. I say it is the opposite. We witness a lot, we meet people in all kinds of situations, when they are at most vulnerable, when they are between life or death, when they don't want to live any more, when they are severely beaten…and that could happen to us all. Everyone. Life is not always clean and tidy, not always fair. You realize that after some years. We can laugh about it, together, about things that are not really fun for anyone else. Undramatized it. We live today, but you never know about tomorrow… You learn that within the ambulance services. I think it is good. You need that reminder sometimes. And I live a richer life thanks to that."

However, sometimes, the burden of the experiences from the major event was too hard to endure, and the recovery process did not end with a positive development. In such situations, the nurses felt overwhelmed and unsuccessful, which led to persistent physical or mental health problems and in some cases, an ending of their career.

> "My colleague, she was incident commander in an accident where two children died. She left the ambulance services after that. She just cried as soon as she came to the work. It was very hard to see her, actually. She was a very competent ambulance nurse, but this crushed her. I tried, we tried all of us. But she took it very personally, she withered away…"

The risk for such outcomes was experienced as higher if the nurse was unexperienced and lacked proper formal or mental preparedness for being faced with the complexity and demands of a major incident.

DISCUSSION

For peer review only

Being deployed as an ambulance nurse in a major incident led physical- and psychological health problems and feelings of being exhausted and vulnerable. To recover, the ambulance nurses took responsibility and strived to find strategies to gain distance from the situation and to create supportive conditions. If successful, the recovery process led to professional and personal growth. However, being deployed in major incidents without significant preparedness or experience could harm individuals and, in the worst case, end their career.

In this study, no serious physical injuries were reported, but despite that, many participants described physical health problems and exhaustion. Although physical well-being has been found to be an important mediator for psychological wellbeing [8], the physical status of ambulance nurses is important when discussing recovery after working in stressful situations.

The nurses demonstrated a high level of awareness of the occurrence of stress and the risks for being traumatized but also self-knowledge and strategies to manage distress. At the same time, several expressed worries about colleagues and sometimes fear of being exposed to situations that might overload their capacity to recover. A realistic appraisal and active decision-making on how to cope with the situation can contribute to resilience after stressful events [7]. In this study, the participants described different activities that can be seen as "rituals", striving for balance concerning professional boundaries between themselves as a person and being an ambulance personnel. It seems that these "rituals" also mediated a transition from "being in the major incident" to "being after the incident" and moving to a more ordinary everyday life. In the theoretical framework of transition [14], this process has been described as a passage from one phase, condition or status to another or as a period between relative stable phases. The transition process is characterised by disconnecting with previous contexts and believes, moving through a period of unfamiliarity and finally the replacement of an existing set of expectations with new ones[15]. The transition, as well as resilience, is depended not only on the individual, but on the social contexts. For a successful transition process, the person needs to have awareness of both their situation and the transition process, and the level of engagement is essential. To develop skills for transition, both supportive measures such as mentorship has been suggested, but also, being exposed to regular transitioning produces prepare individuals to cope with challenges[15]. Future studies are suggested to explore the relationship between transition and resilience among health professionals exposed to potentially traumatic events.

The participants in this study expressed a strong commitment and convention that they were accountable themselves for creating their own recovery process. Being aware of one's own responsibility for one's well-being and using potential traumatic events and severe stress as a foundation to learn and grow from [8] can be applied to the results of this study. All transitions or changes also require time [13], which was strongly emphasized by the study participants who expressed that they sometimes were surprised that their own recovery took longer than expected.

Self-care has been found to be a key to protect against negative health impacts [16]. The study also confirmed that ambulance nurses need to acquire a unique set of personal qualities to promote self-care and self-resilience in their everyday work [17]. A question raised was whether the competence to develop self-care strategies and use experiences to grow is something that can developed over time or whether it is personal attribute needed to be comfortable in a profession such as being a nurse within the ambulance services. Is there a self-selection component that can support resilience within the ambulance services, or is it a result of a professional development process over time?

For peer review only

To mitigate the negative health effects and promote physical and mental wellbeing requires an awareness of the demands as well as the recovery process. Prevention of work-related traumatic stress should rely on a sound psychosocial work environment, systematic training of employees, social support from colleagues and managers, and proper follow-up of employees after a critical event [18]. Well-functioning supportive structures and working environment have been suggested to compensate for short professional experiences [16] and might therefore be of extra importance for less experienced ambulance personnel. Social support seems particularly powerful to reduce negative outcomes of stress [16 19 20]. In line with evidence, organized sessions with the aim to process emotions in the immediate aftermath of the potentially traumatic event, such as debriefings or group counselling, were not experiences that contributed to recovery or resilience. In accordance with previous evidence, such interventions should therefore not be part of the organizational support systems after being deployed to major incidents [21 22]. Instead, this study emphasizes the employer's responsibility to understand the recovery process, to enable time and space for the recovery, and to systematically and with empathy follow up on both physical and mental health, both in the acute phase and over time. Ensuring that ambulance personnel are "fit for duty", in terms of being physically and mentally ready to respond to challenging situations and also being prepared to recover after such situations, after being deployed in major incidents should therefore be a shared responsibility between the individual professional and the employer as a natural component in a resilient ambulance service both in the every day services and in major incidents. Also, further scientific interest in what demands that are required to respond to such incidents and how such qualities develop is needed.

Limitations

As in all qualitative research studies, it can never be excluded that another study sample, the characteristics of the major events forming the basis of this study, or the timing of the data collection might have influenced the results. However, the study sample size was considered to have information power [23], influenced by recruiting study participants with quite recent experiences, high-quality dialogue during the interviews, the two-session interviews and the use of a systematic analysis methodology. None of the included nurses reported worrisome levels of traumatic stress according to the screening. However, it may be that nurses who have left the ambulance services following a potentially traumatic experiences were less likely to be included in this study, given the recruitment procedure, and this must be considered as a limitation.

The two-session interview design was chosen to ensure that all data were collected and to ensure the well-being of the study participants. The second interviews were in general shorter than the first ones but still contributed to rich data by confirming the experiences that were expressed in the first interviews and to verifying the researchers´ interpretations of the first interview statements. No new topics were expressed in the second interviews. All data were analysed and confirmed by researchers with different perspectives on the topic, including an extensive preunderstanding of the ambulance services, major incidents and caring in emotionally challenging situations, which was found to contribute to the understanding of the data.

CONCLUSION

The ambulance nurses demonstrated a high level of awareness of the occurrence of stress and individual responsibility to find strategies to recover. A successful recovery from the physical and

For peer review only

mental exhaustion experienced after being deployed in a major incident required both individual abilities and self-care strategies as well as a supportive working environment. Organized sessions such as debriefings or group counselling did not contribute to recovery and should therefore not be part of the routine organizational support systems. Instead, supporting individual recovery strategies and following up on physical and mental well-being both in the acute phase and over time should be part of all ambulance services procedures after major incidents to promote individual and organizational resilience. Since the resilience of ambulance personnel is essential for a well-functioning emergency services, further research on how to promote such resilience is strongly needed.

List of abbreviations

Post-traumatic stress disorder (PTSD)

Posttraumatic Checklist-Civilian (PCL)

Funding

This research received no specific grant from any funding agency in the public, commercial or not-for-profit sectors.

DECLARATIONS

Ethics approval and consent to participate
The study was reviewed and approved by the Swedish Ethical Review Authority (ref id 2018:232).

Availability of data and materials
The data analysed during the current study are not publicly available due to Swedish laws on research ethics and the ethical approval provided by the Swedish Ethical Review Authority.

Competing interests
The authors declare that they have no competing interests.

Author contributions
KB: Conceptualization, analysis, writing of the manuscript, KH: Conceptualization, data collection, writing of the manuscript. Both authors read and approved the final manuscript.

Acknowledgement
The authors would like to thank Dr Samuel Edelbring for his valuable contribution to the initial design and planning of the data collection for this study.

References

1. Halpern J, Maunder RG, Schwartz B, et al. Identifying, Describing and Expressing Emotions After Critical Incidents in Paramedics. *Journal of Traumatic Stress* 2012;25:111-14.
2. Lawn S, Roberts L, Willis E, et al. The effects of emergency medical service work on the psychological, physical, and social well-being of ambulance personnel: a systematic review of qualitative research. *BMC Psychiatry* 2020;20(1):348.

For peer review only

3. Berger W, Coutinho ES, Figueira I, et al. Rescuers at risk: a systematic review and meta-regression analysis of the worldwide current prevalence and correlates of PTSD in resuce workers. *Social Psychiatry and Psychiatric Epidemiology* 2012;47(6):1001-11.
4. Skogstad L, Heir T, Hauff E, et al. Post traumati stress among rescue workers after terror attacks in Norway. *Occupational and Enivornmental Medicine* 2016;66(7):528-35.
5. Smith EC, Holmes L, Burkle FM. The Physical and Mental Health Challenges Experienced by 9/11 First Responders and Recovery Workers: A Review of the Literature. *Prehospital and Disaster Medicine* 2019 Dec;34(6):625-631.
6. Petrie K, Milligan-Saville J, Gayed A, et al. Prevalence of PTSD and common mental disorders amongst ambulance personnel: a systematic review and meta-analysis. *Social Psychiatry and Psychiatric Epidemiology* 2018;53(9):897-909.
7. Southwick SM, Bonanno GA, Masten AA, et al. Resilience definitions, theory, and challenges: interdisciplinary perspectives. *European Journal of Psychotraumatology* 2014;1(5):25338.
8. Southwick SM, Charney DS. Resilience. The Science of Mastering Life´s Greatest Challanges. Second ed.New York: Cambridge University Press 2018.
9. Shakespeare-Finch J, Daley E. Workplace belongingness, distress, and resilience in emergency service workers. *Psychological Trauma: Theory, Research, Practice, and Policy* 2017;9(1):32-35.
10. Hugelius K, Edelbring S, Blomberg K. Prehospital major incident management: how do training and reallife situations relate? A qualitative study. BMJ Open 2021;11:e048792.
11. Read BL. Serial Interviews: When and Why to Talk to Someone More Than Once. *International Journal of Qualitative Methods* 2018; 17.(1).
12. Braun V, Clarke, V. Using thematic analysis in psychology. *Qualitative Research in Psychology* 2006;3(2):77-101.
13. Arnberg F, Michel PO, Johannesson KB. Properties of Swedish Posttraumatic Stress Measures after a Disaster. *Journal of Anxiety Disorders* 2014;28(4):402-09.
14. Meleis AI, Sawyer LM, Im EO, et al. Experiencing transitions: an emerging middle-range theory. *Advancec in Nursing Science* 2000;23(1):12-28.
15. Al-Yateem N, Docherty, C. Transition: A concept of significance to nursing and health care professionals. *Journal of Nursing Education and Practice*. 2015;5(5):35-40
16. Smith E, Walker T, Burkle FM. Lessons in Post-Disaster Self-Care From 9/11 Paramedics and Emergency Medical Technicians. *Prehospital and Disaster Medicine* 2019;34(3):335-39.
17. Clompus SR, Albarran JW. Exploring the nature of resilience in paramedic practice: A psycho-social study. *International Emergency Nursing* 2016;28:1-7.
18. Skogstad L, Skorstad, M., Lie, A., Conradi, H.S., Heir, T., Weisaeth, L. Work-related post- traumatic stress disorder. *Occupational Medicine* 2013;63(3):175-82.
19. Behnke A, Rojas R, Karrasch S, et al. Deconstructing Traumatic Mission Experiences: Identifying Critical Incidents and Their Relevance for the Mental and Physical Health Among Emergency Medical Service Personnel. *Frontiers in Psychologt* 2019;10:2305.
20. Arnberg F, Hultman CM, Michel M, et al. Social Support Moderates Posttraumatic Stress and General Distress After Disaster. *Journal of Traumatic Stress* 2012;25(6):721-27.
21. Rose SC, Bisson J, Churchill R, et al. Pshycological debriefing for preventing post traumatic stress disorder (PTSD) (Review). Cochrane Database Syst Rev: The Cochrane Library, 2006.
22. International Society for Traumatic Stress Studies. Posttraumatic Stress Disorder Prevention and Treatment Guidelines. Methodology and Recommendations. 2019. Avalibe at: https://istss.org/getattachment/Treating-Trauma/New-ISTSS-Prevention-and-Treatment-Guidelines/ISTSS_PreventionTreatmentGuidelines_FNL.pdf.aspx. Accessed: January 09, 2023.
23. Malterud K, Siersma VD, Guassora AD. Sample Size in Qualitative Interview Studies: Guided by Information Power. *Qualitative Health Reseach* 2016;26(13):1753-60.

For peer review only - http://bmjopen.bmj.com/site/about/guidelines.xhtml

For peer review only

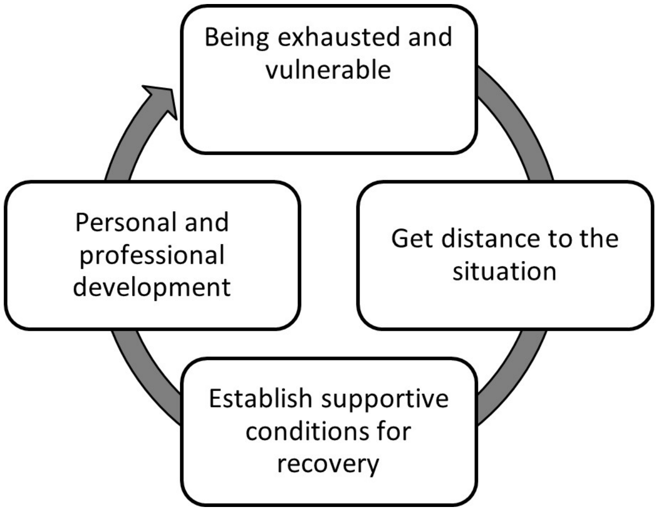

Figure 1. The process of recovery after deployment in a major incident.

107x54mm (330 x 330 DPI)

COREQ (COnsolidated criteria for REporting Qualitative research) Checklist

A checklist of items that should be included in reports of qualitative research. You must report the page number in your manuscript where you consider each of the items listed in this checklist. If you have not included this information, either revise your manuscript accordingly before submitting or note N/A.

Topic	Item No.	Guide Questions/Description	Reported on Page No.
**Domain 1: Research team and reflexivity**			
Personal characteristics			
Interviewer/facilitator	1	Which author/s conducted the interview or focus group?	
Credentials	2	What were the researcher's credentials? E.g. PhD, MD	
Occupation	3	What was their occupation at the time of the study?	
Gender	4	Was the researcher male or female?	
Experience and training	5	What experience or training did the researcher have?	
Relationship with participants			
Relationship established	6	Was a relationship established prior to study commencement?	
Participant knowledge of the interviewer	7	What did the participants know about the researcher? e.g. personal goals, reasons for doing the research	
Interviewer characteristics	8	What characteristics were reported about the inter viewer/facilitator? e.g. Bias, assumptions, reasons and interests in the research topic	
**Domain 2: Study design**			
Theoretical framework			
Methodological orientation and Theory	9	What methodological orientation was stated to underpin the study? e.g. grounded theory, discourse analysis, ethnography, phenomenology, content analysis	
Participant selection			
Sampling	10	How were participants selected? e.g. purposive, convenience, consecutive, snowball	
Method of approach	11	How were participants approached? e.g. face-to-face, telephone, mail, email	
Sample size	12	How many participants were in the study?	
Non-participation	13	How many people refused to participate or dropped out? Reasons?	
Setting			
Setting of data collection	14	Where was the data collected? e.g. home, clinic, workplace	
Presence of non-participants	15	Was anyone else present besides the participants and researchers?	
Description of sample	16	What are the important characteristics of the sample? e.g. demographic data, date	
Data collection			
Interview guide	17	Were questions, prompts, guides provided by the authors? Was it pilot tested?	
Repeat interviews	18	Were repeat inter views carried out? If yes, how many?	
Audio/visual recording	19	Did the research use audio or visual recording to collect the data?	
Field notes	20	Were field notes made during and/or after the inter view or focus group?	
Duration	21	What was the duration of the inter views or focus group?	
Data saturation	22	Was data saturation discussed?	
Transcripts returned	23	Were transcripts returned to participants for comment and/or	

For peer review only

Topic	Item No.	Guide Questions/Description	Reported on Page No.
		correction?	
**Domain 3: analysis and findings**			
Data analysis			
Number of data coders	24	How many data coders coded the data?	
Description of the coding tree	25	Did authors provide a description of the coding tree?	
Derivation of themes	26	Were themes identified in advance or derived from the data?	
Software	27	What software, if applicable, was used to manage the data?	
Participant checking	28	Did participants provide feedback on the findings?	
Reporting			
Quotations presented	29	Were participant quotations presented to illustrate the themes/findings? Was each quotation identified? e.g. participant number	
Data and findings consistent	30	Was there consistency between the data presented and the findings?	
Clarity of major themes	31	Were major themes clearly presented in the findings?	
Clarity of minor themes	32	Is there a description of diverse cases or discussion of minor themes?	

Developed from: Tong A, Sainsbury P, Craig J. Consolidated criteria for reporting qualitative research (COREQ): a 32-item checklist for interviews and focus groups. *International Journal for Quality in Health Care*. 2007. Volume 19, Number 6: pp. 349 – 357

Once you have completed this checklist, please save a copy and upload it as part of your submission. DO NOT include this checklist as part of the main manuscript document. It must be uploaded as a separate file.

For peer review only
